# A Metal‐Polyphenol Self‐Assembled Coated 3D Sponge With Mild Photothermal Antibacterial, Exudate‐Controlling, and Immunomodulatory Functions for Hemostasis and Chronic Wound Healing

**DOI:** 10.1002/advs.77534

**Published:** 2026-09-08

**Authors:** Ziwei Zhang, Feng Qin, Xiaomeng Zhang, Xueqin Shuang, Jiahua Zhou, Ming Cheng, Baicheng Lu, Mingyue Song, Yuntian Bai, Dianying Zhang, Ming Li, Yanhua Wang

**Affiliations:** ^1^ Department of Orthopedics and Trauma Peking University People's Hospital Beijing China; ^2^ Key Laboratory of Trauma and Neural Regeneration Ministry of Education Peking University Beijing China; ^3^ National Center for Trauma Medicine Beijing China; ^4^ Trauma Medicine Center Peking University People's Hospital Beijing China

**Keywords:** 3D nanofiber sponge, chronic wound healing, exudate control, hemostasis, immunomodulation, mild photothermal antibacterial

## Abstract

Chronic wounds represent a prominent clinical challenge, often characterized by impaired healing due to uncontrolled bleeding, persistent bacterial infections, and an imbalanced inflammatory microenvironment. Herein, we report the design and fabrication of a bioinspired, multifunctional 3D nanofiber sponge (TB@CuPNS) armored with a copper‐chelated polydopamine (PDA) coating and functionalized with thrombin (TB). Structurally, the porous sponge demonstrated excellent mechanical resilience, strong exudate absorption capacity, and rapid hemostatic efficiency, as validated in rat liver and femoral artery hemorrhage models. Functionally, the PDA coating imparts mild NIR‐responsive photothermal properties (approximately 45°C) to the sponge, which worked synergistically with pH‐ and ROS‐responsive copper ion release to deliver broad‐spectrum antibacterial and antibiofilm effects through photothermal therapy (PTT) and chemodynamic therapy (CDT). Furthermore, copper ions and the PDA coating cooperatively scavenged excess reactive oxygen species (ROS) and modulated macrophage polarization from an M1 to an M2 phenotype. In an *S. aureus*‐infected diabetic mouse wound model, TB@CuPNS under NIR irradiation significantly accelerated wound repair by stimulating angiogenesis, increasing collagen deposition, and alleviating chronic inflammation. This study provides an effective strategy for fabricating advanced biomaterials that actively regulate the complex biological cascades involved in wound repair.

## Graphical abstract

A thrombin‐functionalized copper‐polydopamine nanofiber sponge integrates rapid hemostasis, mild photothermal antibacterial therapy, stimulus‐responsive Cu^2^^+^ release, chemodynamic oxidative stress, and macrophage immunomodulation. This multifunctional dressing suppresses infection, regulates inflammation, promotes angiogenesis and re‐epithelialization, and accelerates the repair of chronic wound healing under NIR activation.

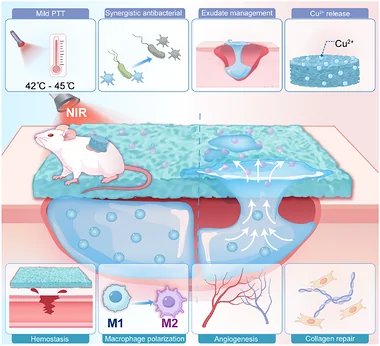

## 1 Introduction

Chronic wounds, characterized by impaired healing progression due to unresolved and sustained inflammation, pose a significant global healthcare challenge [1, 2]. The native wound healing cascade is a precisely orchestrated biological process comprising four sequential yet overlapping stages: hemostasis, inflammation, proliferation, and tissue remodeling [3, 4, 5]. Impairment at any phase disrupts this highly ordered sequence, culminating in wound chronicity. The initial hemostasis stage is crucial for preventing excessive bleeding and forming a temporary fibrin matrix that supports subsequent cell recruitment and activity [6]. Failure to achieve rapid and effective hemostasis leads to prolonged inflammation and fosters a microenvironment conducive to bacterial colonization, thereby perturbing the entire tissue repair cascade [7]. This pathological state is further exacerbated by the dysregulated microenvironment, which is common in conditions such as diabetes or venous ulcers, characterized by high oxidative stress, persistent bacterial infections, and impaired angiogenesis [8]. The increasing prevalence of antibiotic‐resistant bacterial strains further complicates clinical management. Therefore, advanced wound management strategies should aim to re‐establish an ordered healing cascade by simultaneously achieving rapid hemostasis, infection control, inflammatory regulation, and tissue regeneration.

To address these multifaceted challenges, a diverse array of advanced wound dressings, including hydrogels, films, and sponges, has been engineered [9, 10]. A crucial design consideration for chronic wound dressings is the coordinated management of initial hemorrhage and subsequent exudate accumulation [11]. Prolonged or uncontrolled bleeding can directly initiate a persistent inflammatory cascade and increase the risk of bacterial colonization, both of which are major pathological drivers of wound chronicity [12]. Meanwhile, excessive wound exudate provides a nutrient‐rich environment for microbial proliferation. In addition, high levels of proteolytic enzymes degrade the nascent extracellular matrix (ECM) and adjacent healthy tissue, further hindering tissue repair [13]. Therefore, an ideal chronic wound dressing should exhibit multiple functions, including rapid hemostatic capacity, potent antibacterial activity, efficient exudate management while maintaining a moist environment, and unimpeded gas exchange [14]. Among emerging biomaterial platforms, three‐dimensional (3D) nanofiber sponges fabricated via electrospinning and related techniques have attracted considerable research attention [15, 16]. The lightweight structure, high porosity, superior liquid absorption capacity, and ECM‐mimetic architecture  make them promising scaffolds for advanced wound repair and efficient loading of bioactive therapeutic agents [17].

Photothermal therapy (PTT) has been extensively used to eradicate bacteria and biofilms efficiently through localized hyperthermia [18, 19]. However, uncontrolled or excessive heat accumulation during conventional PTT may cause collateral thermal injury to surrounding healthy tissues and disrupt the wound microenvironment, thereby compromising tissue repair [18, 20]. Therefore, precise thermal control is particularly important for infected wound treatment. In this study, a local temperature of approximately 45°C was selected as a mild photothermal window to balance antibacterial efficacy and tissue compatibility. Mild hyperthermia at this level can enhance bacterial membrane permeability and antibacterial susceptibility, while reducing the risk of collateral thermal injury associated with PTT at higher temperatures [21, 22, 23]. In addition, local mild heating has been reported to increase wound perfusion and oxygen tension, which may further support tissue repair in infected wounds [20, 24]. Polydopamine (PDA), a naturally occurring melanin analog, is widely used in antibacterial and antitumor treatments owing to its versatile characteristics, including facile synthesis, antioxidant activity, strong adhesion, biocompatibility, and efficient photothermal conversion [25, 26, 27]. Crucially, its catechol‐rich structure provides abundant sites for chelating metal ions, making it a versatile platform for integrating additional therapeutic functions [28].

PDA inherently enables the chelation and incorporation of therapeutic metal ions, including copper ions (Cu^2^^+^), an essential trace element for orchestrating efficient wound healing [29]. As an established cofactor for enzymes critical to angiogenesis and collagen deposition, Cu^2^^+^ also exhibits intrinsic broad‐spectrum antibacterial activity [30, 31, 32]. Furthermore, Cu^2^^+^ can initiate a potent antimicrobial cascade via chemodynamic therapy (CDT) [26], a strategy that has shown promising antibacterial efficacy by catalyzing the generation of cytotoxic hydroxyl radicals (·OH) from hydrogen peroxide (H_2_O_2_). Conventional CDT commonly relies on ferrous ions (Fe^2^^+^) from iron‐containing nanomaterials to drive this Fenton reaction [33], while recent studies have confirmed nanoscale copper peroxide (n‐CuO_2_) as a highly attractive CDT candidate with excellent Fenton‐like catalytic activity [34]. This dual antibacterial mechanism, which integrates copper‐mediated CDT into a PDA‐coated scaffold, offers a synergistic therapeutic platform that combines mild photothermal therapy (PTT) with controllable chemodynamic therapy for comprehensive chronic wound management.

**SCHEME 1:**
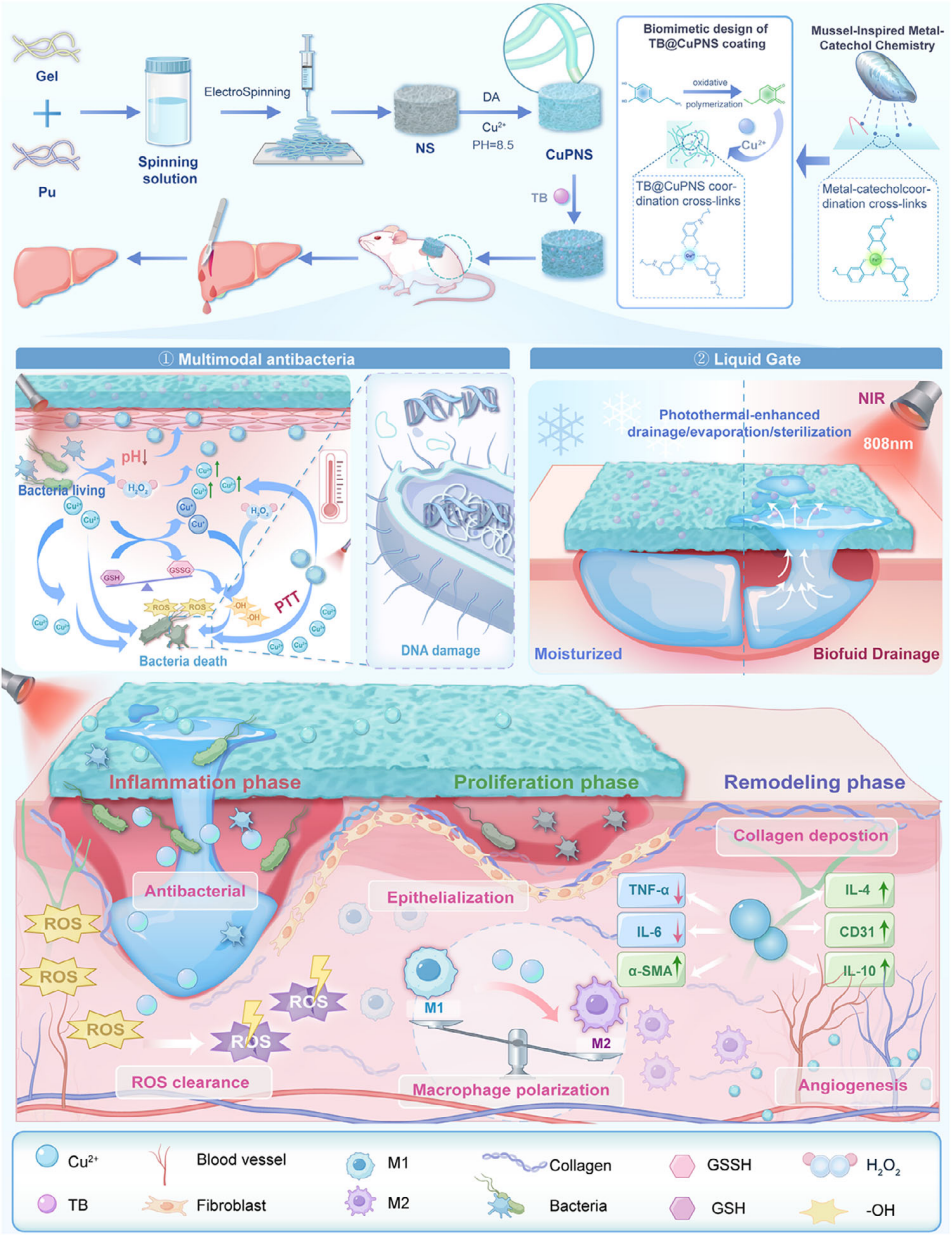
Schematic illustration of the construction and therapeutic mechanism of TB@CuPNS for infected chronic wound healing. The porous 3D nanofiber sponge is engineered through self‐assembly between Cu^2^^+^ and polyphenols, followed by thrombin functionalization, endowing the sponge with rapid hemostatic activity, exudate regulation, NIR‐responsive mild photothermal antibacterial performance, and stimuli‐responsive Cu^2^^+^ release. Through localized infection control and remodeling of the pathological wound microenvironment, TB@CuPNS alleviates excessive inflammation, modulates macrophage polarization, promotes angiogenesis, collagen deposition, and re‐epithelialization, and ultimately accelerates wound repair.

Here, we designed a multifunctional dynamic nanofiber sponge TB@CuPNS, armored with a copper‐chelated polydopamine (PDA) coating and functionalized with thrombin (TB) for chronic wound therapy (Scheme 1). The 3D sponge provides an ECM‐mimetic porous structure for exudate management and tissue integration. Thrombin confers rapid hemostatic activity, while the Cu‐PDA coating enables NIR‐responsive mild photothermal heating, pH/ROS‐responsive Cu^2^^+^ release, broad‐spectrum antibacterial and antibiofilm activity, ROS regulation, and macrophage immunomodulation. By coupling hemostasis, infection control, exudate management, and immune regulation within one topical scaffold, TB@CuPNS provides a coordinated strategy for remodeling the pathological wound microenvironment and promoting chronic wound repair.

## 2 Results and Discussion

### 2.1 Synthesis and Physicochemical Characterization of TB@CuPNS Sponge

Wound healing represents a dynamic and complex physiological process. An ideal wound dressing should integrate multifunctional properties, including efficient hemostasis, outstanding antibacterial activity, and favorable biocompatibility, to collectively promote wound repair. To address these clinical demands, we initially converted electrospun PU/gelatin nanofibers into a robust 3D sponge architecture. This foundational sponge was then endowed with multifunctionality via a bioinspired, one‐step metal‐polyphenol self‐assembly strategy, thereby creating a bioactive coating on the nanofiber surfaces. Finally, this armored scaffold was further functionalized by immobilizing thrombin (TB), ultimately forming the TB@CuPNS composite system.

**FIGURE 1:**
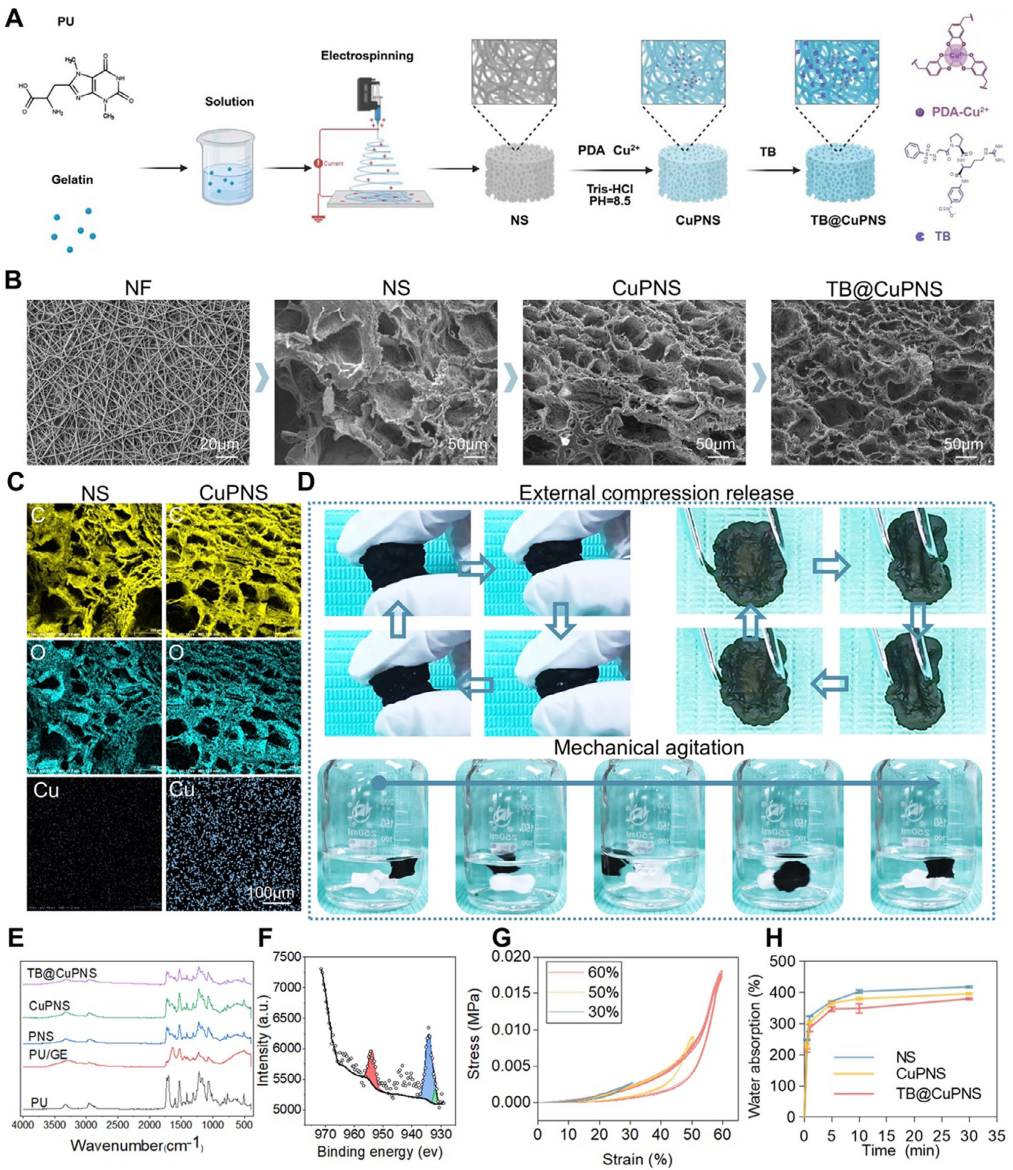
Synthesis and characterization of TB@CuPNS. (A) Schematic illustration of the synthesis process of TB@CuPNS. (B) Representative scanning electron microscopy (SEM) images of the sponges at different magnifications. (C) Energy‐dispersive x‐ray spectroscopy (EDS) elemental mapping images of NS and CuPNS. (D) Morphological changes of TB@CuPNS during external compression and release, along with evaluation of its mechanical stability under intense agitation. (E) Fourier‐transform infrared spectroscopy (FTIR) spectra of different sponges. (F) X‐ray photoelectron spectroscopy (XPS) spectra of TB@CuPNS. (G) Compressive stress‐strain curves of TB@CuPNS. (H) Water absorption rates of NS, CuPNS, and TB@CuPNS. All statistical data are represented as mean ± SD, *n* = 3 independent experiments per group.

The synthesis pathway is schematically illustrated in Figure 1A. The key steps involved: (i) fragmentation and homogenization of the initial 2D nanofiber mat; (ii) formation of a stable three‐dimensional nanofiber sponge (NS) through freeze‐drying and cross‐linking; (iii) immersion of the NS scaffold in a solution containing dopamine and Cu^2^^+^, and through the synchronous reaction of dopamine self‐polymerization and metal ion chelation, the in situ formation of copper‐chelated polydopamine coating (CuPNS) was achieved; (iv) final functionalization via thrombin (TB) immobilization to obtain TB@CuPNS.

The macroscopic appearance and microstructural morphology of the sponges at each preparation stage were systematically characterized using digital photographs and scanning electron microscopy (SEM) (Figure 1B). The images revealed that the initial 2D nanofiber nonwoven mat (NF) was successfully transformed into a 3D sponge with a highly porous, interconnected architecture. Importantly, this porous architecture was preserved through the CuPNS and TB@CuPNS functionalization processes, which is crucial for wound exudate absorption, gas exchange, and cellular infiltration. Statistical analysis further confirmed the uniformity of the fiber diameters across different groups (Figure S1). Notably, the deposition of CuPNS and TB@CuPNS did not destroy the ECM‐like reticular fiber structure of the fiber scaffold. The gradual increase in fiber diameter from NS to CuPNS and TB@CuPNS further verified the successful deposition of PDA and thrombin on the NS surface. In contrast, the average fiber diameter showed no obvious difference between NF and NS, indicating that the 3D formation process did not alter the intrinsic fiber morphology.

The successful and homogeneous incorporation of the Cu‐PDA coating was validated by EDS elemental mapping, which showed a uniform distribution of copper (Cu) alongside carbon (C) and oxygen (O) in the CuPNS scaffold, in contrast to the NS control (Figure 1C). Fourier‐transform infrared spectroscopy (FTIR) analysis (Figure 1E) further confirmed the presence of characteristic peaks of the PU/gelatin matrix and the PDA coating in the functionalized CuPNS and TB@CuPNS sponges. Specifically, high‐resolution XPS spectra of the Cu 2p region provided clear evidence of Cu presence on the surfaces of CuPNS and TB@CuPNS sponges (Figure 1F), with characteristic peaks corresponding to Cu^2^^+^ observed, further confirming the successful polymerization of dopamine and chelation with Cu^2^^+^.

The mechanical and physical properties of the sponges were then systematically evaluated. The sponges exhibited excellent mechanical resilience, as demonstrated by rapid shape recovery after manual compression and maintained structural integrity during vigorous mechanical agitation in aqueous solution (Figure 1D). Wettability and water absorption capacity are key factors affecting cell activity and tissue regeneration [35]. Highly absorbent scaffolds can efficiently manage wound exudate, reduce the risk of bacterial infection, and maintain a moist microenvironment favorable for wound healing [36]. The TB@CuPNS sponge absorbed water quickly upon contact and exhibited good elasticity, with nearly complete recovery to its initial cylindrical shape after external compression (Figure 1D and Video S1).

Nevertheless, differences in water absorption capacity and expansion rate were observed across scaffold groups. The NS sponge showed the highest water absorption and swelling performance, followed by CuPNS and TB@CuPNS (Figure 1H), mainly due to differences in porosity among the samples. Nevertheless, all sponges maintained a high porosity of over 50% (Figure S3), indicating that the functional coatings were conformally applied without occluding the crucial porous network required for fluid exchange and cellular infiltration. Furthermore, TB@CuPNS maintained stable morphology and structure even under vigorous mechanical stirring (Video S2), further confirming its structural stability.

Quantitative tensile tests confirmed the robust mechanical properties of TB@CuPNS (Figure 1G). The Young's modulus was retained at 0.0284 MPa after 20 consecutive cyclic loading tests, reflecting only a minor reduction from the initial 0.0293 MPa (Figure S2). These mechanical features are particularly crucial for dressings applied to dynamic wound sites, as they ensure reversible water uptake and stable shape recovery even under repetitive external compression (Video S3), which is essential for dressings used in mechanically active wound environments.

In conclusion, the TB@CuPNS composite sponge was successfully prepared by sequentially assembling different functional elements via a straightforward and scalable strategy. The comprehensive characterization results confirmed the successful synthesis, uniform functionalization, excellent mechanical stability, and favorable physical properties of TB@CuPNS. These intrinsic bioactivities and structural advantages support its potential application in chronic wound repair.

### 2.2 Photothermal Performance and Stimuli‐Responsive Cu^2^^+^ Release Behavior

**FIGURE 2:**
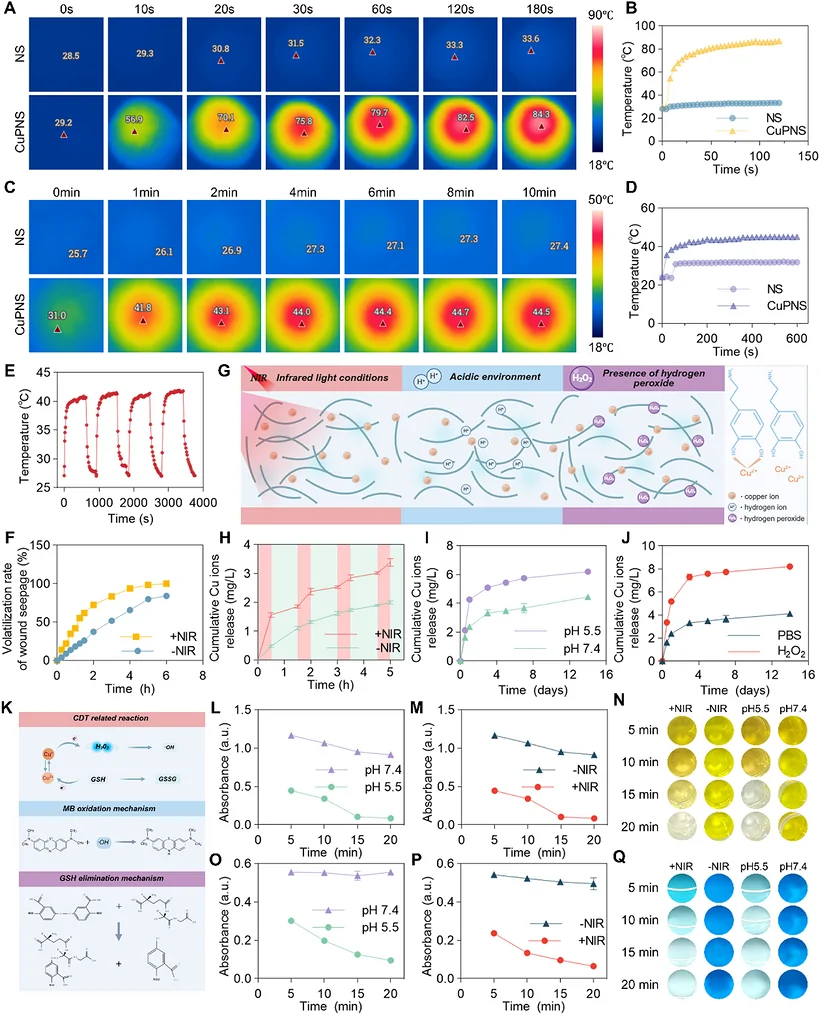
Photothermal performance and Cu^2^^+^ release behavior of TB@CuPNS. (A) Infrared thermal images of NS and CuPNS under 808 nm NIR irradiation in dry conditions for 180 s. (B) Photothermal heating curves of NS and CuPNS in dry conditions during 808 nm NIR irradiation for 180 s. (C) Infrared thermal images of NS and CuPNS under 808 nm NIR irradiation in moist conditions for 10 min. (D) Photothermal heating curves of NS and CuPNS in moist conditions during 808 nm NIR irradiation for 10 min. (E) Recycling heating profiles of CuPNS under NIR irradiation for four cycles. (F) Simulated wound exudate evaporation capacity of CuPNS with and without NIR irradiation. (G) Schematic diagram of stimuli‐responsive Cu^2^^+^ release regulation. (H) Cumulative release of Cu^2^^+^ from CuPNS with and without NIR irradiation. (I) Cu^2^^+^ release from CuPNS at different pH values. (J) Cu^2^^+^ release from CuPNS in the presence or absence of H_2_O_2_. (K) Schematic illustration of the chemodynamic therapy (CDT)‐associated catalytic mechanism and the principles of glutathione (GSH) consumption and methylene blue (MB) degradation assays. (L) GSH consumption under different pH conditions. (M) GSH consumption with and without NIR irradiation. (N) Photographs of glutathione colorimetric reactions from different groups. (O) Methylene blue (MB) degradation under different pH conditions. (P) Methylene blue degradation with and without NIR irradiation. (Q) Representative photographs of MB degradation in different groups. All statistical data are represented as mean ± SD, *n* = 3 independent experiments per group.

Metal‐polyphenol networks (MPNs) formed from transition metal ions and polyphenols have been widely reported to exhibit strong photothermal conversion.  The coordination between metal ions and phenolic hydroxyl groups can generate electron transition pathways that support optical energy absorption under NIR irradiation. Under dry conditions, 808 nm NIR irradiation caused a rapid temperature increase in CuPNS, with the scaffold reaching 84.3°C at 180 s, whereas the uncoated NS scaffold showed only a negligible thermal response (Figure 2A,B). This dry‐state assay was used to confirm the intrinsic photothermal conversion capability of the Cu‐PDA coating rather than to reproduce the hydrated wound environment.

To simulate the physiological microenvironment of chronic wounds, the photothermal performance was also evaluated in moist conditions across various laser power densities (Figure S4). Under this hydrated condition, CuPNS maintained stable heating behavior and reached approximately 44.5°C within 10 min (Figure 2C,D). The lower equilibrium temperature and longer irradiation time compared with the dry‐state assay reflect heat absorption and dissipation by PBS or water, which more closely mimics wound exudate or tissue fluid. Therefore, 0.7 W·cm^−2^ was selected for subsequent antibacterial and in vivo experiments to maintain the wound region within a mild photothermal window of approximately 45°C. This temperature range has been reported to enhance antibacterial susceptibility and bacterial membrane permeability while reducing the risk of collateral thermal injury associated with PTT at higher temperatures, thereby providing a rational balance between antibacterial efficacy and tissue compatibility [22, 23]. The photothermal recyclability of CuPNS was evaluated by four consecutive heating and cooling cycles, during which no obvious attenuation was observed (Figure 2E). These results suggest that CuPNS exhibits stable optical absorption and photothermal conversion properties.

A key feature of 3D wound dressings is their ability to respond actively to the wound microenvironment. The photothermal effect of CuPNS was shown to regulate wound fluid management, demonstrated by the accelerated evaporation rate of simulated wound exudate under NIR stimulation (Figure 2F). Beyond fluid control, the sponge was designed for controlled release of Cu^2^^+^, as shown in Figure 2G. We confirmed that Cu^2^^+^ release was highly responsive to multiple wound‐relevant stimuli. The release was significantly increased by external NIR irradiation (Figure 2H), and the coordination of metal ions and polyphenols was greatly influenced by the inflammatory microenvironment. Compared with neutral pH (7.4), Cu^2^^+^ was released more readily from CuPNS in a weakly acidic pH (5.5), which helps eliminate pathogenic bacteria even without NIR irradiation (Figure 2I). Additionally, the presence of hydrogen peroxide (H_2_O_2_) in infected wounds also boosts Cu^2^^+^ release (Figure 2J). This stimuli‐responsive release behavior allows CuPNS to deliver Cu^2^^+^ to infected wound areas, reducing potential systemic side effects and promoting wound healing.

The CDT‐associated catalytic mechanism of CuPNS was further investigated through GSH depletion and methylene blue (MB) degradation assays (Figure 2K–Q). GSH, a vital component of bacterial antioxidant defenses, can mitigate oxidative stress by regulating the redox state of metal ions. Therefore, GSH depletion was evaluated to determine whether CuPNS could disrupt bacterial redox homeostasis. During the reaction, GSH was oxidized to glutathione disulfide (GSSG), resulting in a color change from yellow to colorless (Figure 2N). As shown in Figure 2L,M, GSH consumption increased as pH decreased, resulting from the catalytic interaction between Cu^2^^+^ released from CuPNS and GSH, forming Cu^+^ and GSSG. Notably, CuPNS treated with NIR irradiation caused the most significant color change of GSH at pH 5.5, indicating pronounced depletion of GSH. Based on these results, we confirmed that the sponge has a strong ability to consume GSH under acidic and mild photothermal conditions, which can disrupt the bacterial antioxidant defense system and enhance bacterial killing via oxidative stress.

Meanwhile, methylene blue (MB) degradation was used to evaluate the Fenton‐like catalytic activity of TB@CuPNS. In this assay, MB serves as a chromogenic probe for ·OH generation, as ·OH can oxidatively degrade MB, leading to fading of its blue color and a decrease in its characteristic absorbance at 664 nm. As illustrated in Figure 2O, MB degradation was enhanced under weakly acidic conditions, indicating that the Fenton‐like catalytic activity of TB@CuPNS was pH‐dependent and more efficient at pH 5.5, which resembles the acidic microenvironment of infected wounds. In addition, NIR irradiation further accelerated MB degradation, suggesting that mild photothermal stimulation enhanced Cu^2^^+^ release and facilitated redox cycling between Cu^2^^+^ and Cu^+^ in the presence of GSH and H_2_O_2_, thereby increasing ·OH generation (Figure 2P). Consistently, visual photographs showed that the MB solution in the TB@CuPNS‐containing groups gradually faded from blue to nearly colorless, whereas the control group without NIR irradiation at pH 7.4 retained a blue color (Figure 2Q). Overall, these results confirm that TB@CuPNS can react with H_2_O_2_ to generate ·OH in a weakly acidic environment, and NIR‐induced mild photothermal stimulation further boosts the catalytic efficiency of TB@CuPNS.

In summary, the TB@CuPNS sponge shows excellent photothermal properties and stability owing to the CuPNS component. The mild local hyperthermia (around 45°C) generated by TB@CuPNS under NIR irradiation is the main driver of mild photothermal therapy (PTT), enabling bacterial killing while avoiding damage to normal tissues and cells. Moreover, TB@CuPNS demonstrates stimuli‐responsive Cu^2^^+^ release triggered by NIR irradiation, acidic pH, and H_2_O_2_, together with effective GSH depletion and enhanced Fenton‐like catalytic activity in a simulated wound microenvironment. This combined strategy of mild PTT, stimuli‐triggered Cu^2^^+^ release, and CDT‐induced oxidative stress provides a multimodal antibacterial mechanism for TB@CuPNS and supports its therapeutic potential for infected chronic wounds.

### 2.3 In Vitro Antibacterial and Antibiofilm Activities of TB@CuPNS

Persistent bacterial infection is a major obstacle to wound healing. Therefore, we thoroughly assessed the antibacterial properties of the TB@CuPNS sponge. To establish a safe and effective therapeutic range, we first examined the biocompatibility of different Cu^2^^+^ concentrations using the CCK‐8 assay, which revealed a gradual decline in cell viability from 96.6% to 47.5% as the Cu^2^^+^ concentration increased from 0.1 to 1.5 mg/mL (Figure S5). These findings indicate that 0.5 mg/mL Cu^2^^+^ provided a suitable balance between antibacterial efficacy and cytocompatibility, maintaining acceptable cell viability while avoiding the increased cytotoxicity observed at 1.0 and 1.5 mg/mL.

**FIGURE 3:**
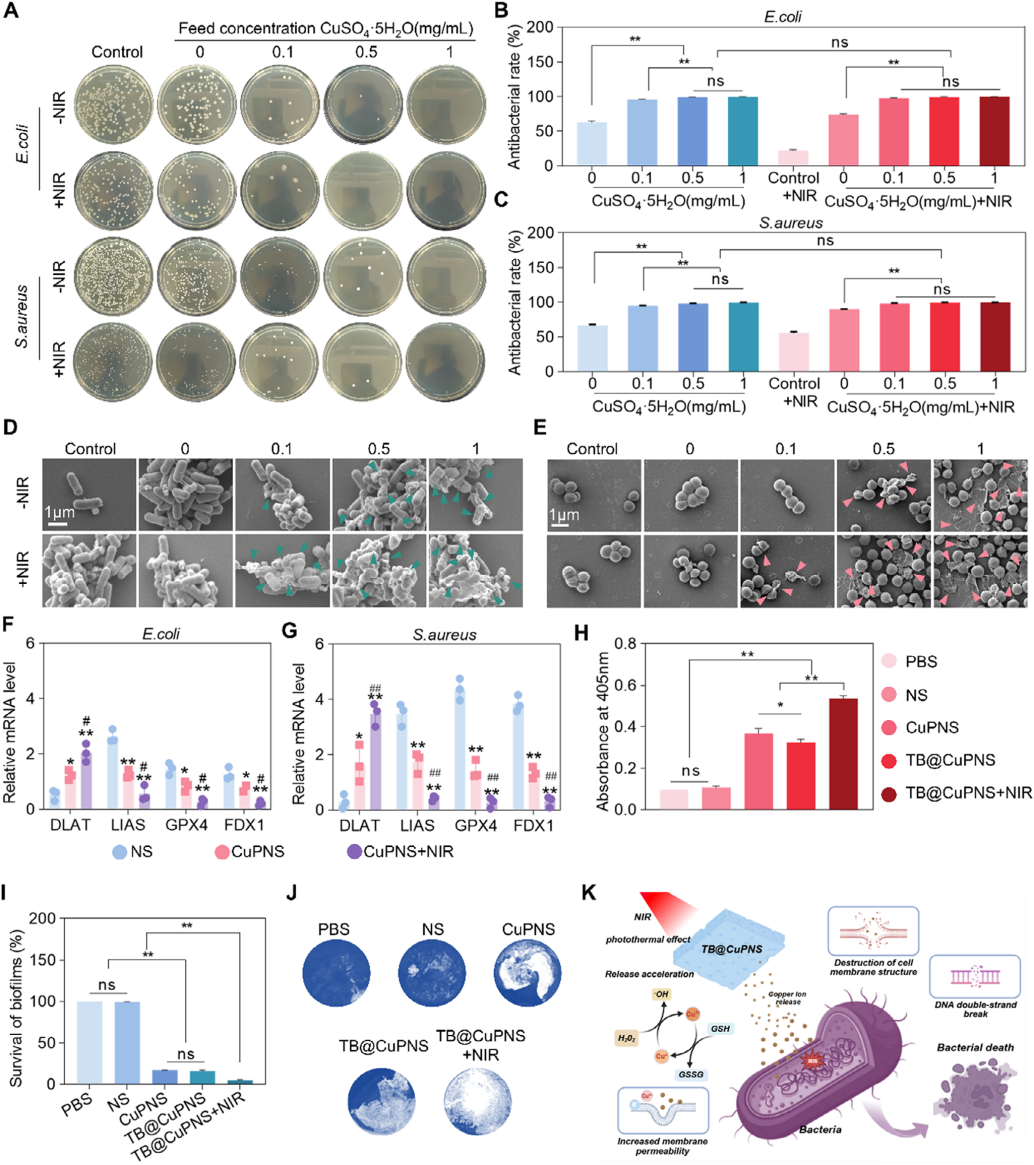
Antibacterial bioactivities of TB@CuPNS. (A) Bacterial colony images of *E. coli* and *S. aureus* after exposure to sponges prepared with different CuSO_4_·5H_2_O chelation concentrations, with or without NIR irradiation. (B,C) Quantitative analysis of bacterial colony counts for *E. coli* and *S. aureus*, respectively. (D,E) SEM images showing morphological changes in *E. coli* (D, green arrows) and *S. aureus* (E, red arrows) after exposure to sponges prepared with different CuSO_4_·5H_2_O chelation concentrations, with or without NIR irradiation. Arrows indicate representative sites of bacterial membrane wrinkling, collapse, and rupture. Scale bar = 1 µm. (F, G) RT‐qPCR analysis of cuproptosis genes (DLAT, LIAS, GPX4, FDX1) in *E. coli* (F) and *S. aureus* (G). (H) Evaluation of bacterial membrane permeability. (I) Quantitative analysis of crystal violet staining. (J) Crystal violet staining images of *S. aureus* and *E. coli* biofilms. (K) Schematic diagram illustrating the antibacterial mechanism of TB@CuPNS under NIR activation. (B, C, F‐I) Statistical analysis was performed using one‐way ANOVA with Tukey's post hoc test. *n* = 3 independent experiments per group. All data in the graphs are presented as mean ± SD; ^*^ *p* < 0.05, ^**^ *p* < 0.01.

TB@CuPNS sponges were then prepared using different CuSO_4_·5H_2_O concentrations during PDA‐assisted chelation. In Figure 3A–C, the concentrations labeled as 0, 0.1, and 0.5 mg/mL refer to the initial CuSO_4_·5H_2_O concentrations used during sponge preparation, rather than the released Cu^2^^+^ concentrations from the final sponges. As the CuSO_4_·5H_2_O chelation concentration increased, both turbidity and CFU counts of *S. aureus* and *E. coli* significantly decreased (Figure 3A). Quantitative analysis (Figure 3B,C) further confirmed that, without near‐infrared light irradiation, the inhibition rates were 95.72% and 99.08% at 0.1 and 0.5 mg/mL CuSO_4_·5H_2_O concentrations, respectively. After NIR irradiation, the bactericidal effect was further enhanced, with inhibition rates of 97.57% and 99.24% at the corresponding concentrations. This synergistic effect is likely due to photothermal‐induced scaffold degradation, which accelerates Cu^2^^+^ release and promotes ROS production through Fenton‐like reactions.

To further confirm the antibacterial mechanism, SEM was used to observe morphological changes in these two bacterial strains. For the control groups, *S. aureus* and *E. coli* displayed smooth surfaces and intact structures, each showing their typical spherical and rod‐shaped morphologies, respectively. In contrast, after exposure to TB@CuPNS under NIR irradiation, both *S. aureus* and *E. coli* showed severe morphological alterations, characterized by extensive wrinkling and collapse of membranes in both *S. aureus* and *E. coli* (Figure 3D,E). These findings indicate that bacterial membrane disruption is a major contributor to the potent antibacterial effect of NIR‐activated TB@CuPNS. Beyond the physical disruption of bacterial membranes, we further investigated the Cu^2^^+^‐mediated oxidative antibacterial mechanism associated with CDT‐like activity.

To investigate the comprehensive antibacterial mechanism, we further examined whether Cu^2^^+^ release combined with NIR irradiation induced a bacterial cuproptosis‐like response. Key cuproptosis‐related genes (FDX1, LIAS, GPX4, and DLAT) were then evaluated in both S. aureus and *E. coli* using RT‐qPCR, with the primer sequences listed in Table S1 [37]. Compared with the NS group, the CuPNS group downregulated FDX1, LIAS, and GPX4 expression, and upregulated DLAT expression in both bacterial strains (Figure 3F). These changes were further strengthened by NIR irradiation (Figure 3G), suggesting that TB@CuPNS + NIR induced a bacterial cuproptosis‐like response rather than simple thermal killing alone. Importantly, NIR irradiation enhanced bacterial membrane damage, as shown by higher bacterial membrane permeability (Figure 3H). The TB@CuPNS + NIR group exhibited the highest membrane permeability (53.78%), which was significantly higher than those of the TB@CuPNS (32.40%), CuPNS (36.71%), and NS (10.68%) groups, thereby facilitating Cu^2^^+^ entry into bacterial cells.

Biofilm eradication efficiency was evaluated using crystal violet (CV) staining (Figure 3J). The TB@CuPNS + NIR group exhibited the best removal effect, with a residual biofilm ratio of 5.01%, which was significantly lower than that of TB@CuPNS (16.39%), CuPNS (17.29%), and NS (99.55%) (Figure 3I). These results demonstrated the potent antibiofilm activity of photothermal TB@CuPNS, in addition to its capacity to combat planktonic bacteria. Based on the superior antibacterial performance of the sponge, we proposed a comprehensive mechanism for TB@CuPNS (Figure 3K). Upon NIR activation, TB@CuPNS produces localized mild hyperthermia and enhances Cu^2^^+^ release, resulting in bacterial membrane rupture, intracellular ROS accumulation, and DNA damage, ultimately leading to bacterial death.

Together, these findings demonstrate that NIR‐activated TB@CuPNS effectively eradicates *E. coli *and *S. aureus*, including established biofilms. Compared with single‐mode high‐temperature photothermal sterilization, the present system combines mild PTT, stimuli‐responsive Cu^2^^+^ release, bacterial membrane disruption, and Cu^2^^+^‐mediated oxidative stress. This integrated antibacterial mechanism enables efficient bacterial killing under lower and more tissue‐compatible thermal conditions, supporting its application in infected chronic wound treatment.

### 2.4 In Vitro Cytocompatibility, Angiogenic Activity, and Cell Behavior Regulation

**FIGURE 4:**
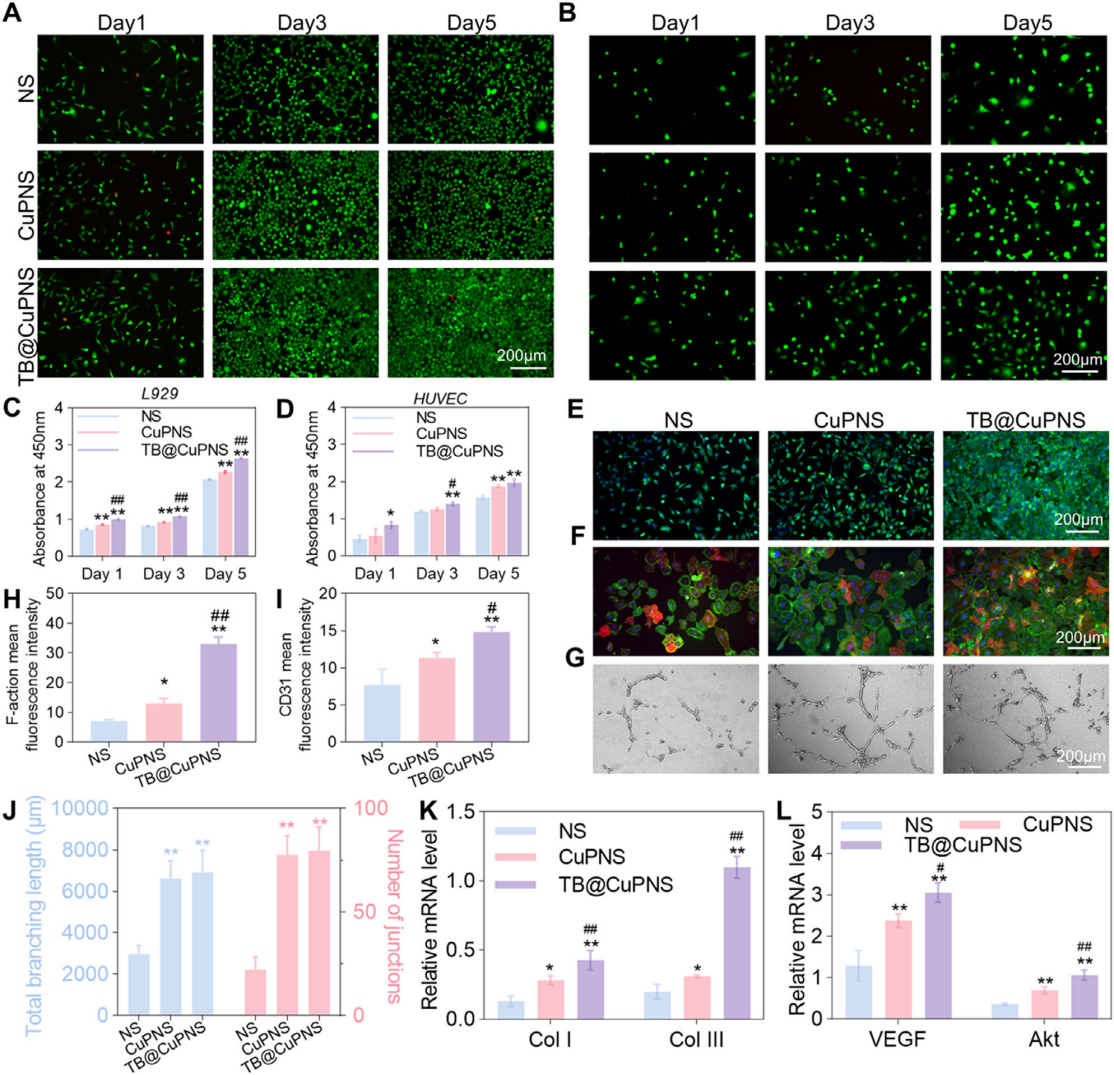
In vitro biocompatibility and cell behavior regulation of TB@CuPNS. (A, B) Live/dead staining of L929 cells (A) and HUVECs (B) after culture with sponge extracts for 1, 3, and 5 days. (C) Cell viability of L929 cells in NS, CuPNS, and TB@CuPNS groups. (D) Cell viability of HUVECs in NS, CuPNS, and TB@CuPNS groups. (E) Immunofluorescence staining of L929 cells after 3 days of culture, with Col I shown in green and nuclei counterstained with DAPI (blue). (F) Immunofluorescence image of HUVECs on day 5 with F‐actin visualized in red, CD31 in green, and nuclei in blue. (G) In vitro tube formation assay of HUVECs. (H) Quantitative analysis of F‐actin fluorescence intensity from immunofluorescence staining. (I) Quantification of CD31 fluorescence intensity from immunofluorescence staining. (J) Quantitative analysis of junction number and total branching length in HUVEC tube networks. (K) Quantitative RT‐qPCR analysis of Col I and Col III mRNA expression in L929 cells. (L) Quantitative RT‐ qPCR analysis of VEGF and Akt mRNA expression in HUVECs. (C, D, H‐L) Statistical analysis was performed using one‐way ANOVA with Tukey's post hoc test. *n* = 3 independent experiments per group. All data in the graphs are presented as mean ± SD; ^*^ *p* < 0.05 and ^**^ *p* < 0.01 vs. the NS group; #p < 0.05 and ##p < 0.01 vs. the CuPNS group.

Wound healing is characterized by the coordinated interplay of multiple biological processes [38]. To evaluate the cytocompatibility and regenerative potential of the TB@CuPNS scaffold, we assessed cell viability, proliferation, migration, collagen‐related matrix deposition, and tube formation in representative cells involved in wound repair, including L929 fibroblasts, HaCaT keratinocytes, and HUVECs. Among all groups, the number of live L929 cells and HUVECs gradually increased with prolonged culture duration, demonstrating the favorable biocompatibility and non‐toxicity of these sponges (Figure 4A,B). Quantitative evaluation of cell viability further confirmed the proliferative capacity of L929 cells and HUVECs in all groups (Figure 4C,D). Consistently, HaCaT keratinocytes also maintained good viability after treatment with different sponges (Figure S6), indicating that TB@CuPNS exhibited favorable compatibility with both dermal and epidermal repair‐related cells. Notably, TB@CuPNS significantly enhanced the proliferation of both L929 cells and HUVECs compared with NS and CuPNS, further supporting its regenerative potential for wound repair.

Fibroblasts function as the primary cellular mediators of Col I and Col III biosynthesis throughout the wound repair process [39]. To evaluate the morphology and collagen synthesis of L929 fibroblasts cultured on the engineered scaffolds, immunofluorescence staining was performed. Quantitative imaging demonstrated that while all scaffold groups supported robust cellular morphology, L929 cells seeded on TB@CuPNS scaffolds displayed markedly elevated Col I deposition relative to both CuPNS and NS groups (Figure 4E). The mRNA expression levels of Col I and Col III were further quantified using RT‐qPCR, with the primer sequences listed in Table S2. TB@CuPNS significantly upregulated both Col I and Col III expression compared with the other groups (Figure 4K), indicating enhanced ECM‐related regenerative activity. Considering that fibroblast migration is essential for granulation tissue formation and wound contraction, L929 cell migration was further evaluated using a scratch wound healing assay. The TB@CuPNS group showed a higher migration rate at 24 and 48 h than the control groups (Figure S7), suggesting that TB@CuPNS supports both fibroblast matrix deposition and migration. Keratinocyte migration is a critical step in re‐epithelialization. Therefore, the migratory behavior of HaCaT cells was further evaluated using a Transwell assay. The assay revealed a greater number of migrated cells in the TB@CuPNS group (Figure S8), indicating that TB@CuPNS also supports keratinocyte migration associated with re‐epithelialization.

VEGF signaling triggers a cascade of endothelial cell processes, such as migration, proliferation, and vascular morphogenesis [40]. After 5 days of culture, cell morphology and spreading were assessed by cytoskeleton and immunofluorescence staining (Figure 4F). HUVECs adhered and spread well on all scaffolds. In particular, those cultured on TB@CuPNS and CuPNS formed more circular connections, a characteristic morphological feature of endothelial cell maturation and formation of vascular‐like structures. Quantification of F‐actin/CD31 immunofluorescence intensity confirmed that HUVECs on TB@CuPNS and CuPNS scaffolds expressed higher levels of CD31 than those on NS scaffolds (Figure 4H,I). Similar results were obtained from the in vitro tube formation assay (Figure 4G). Quantitative analysis further demonstrated that the TB@CuPNS group exhibited a marked increase in junction number and total branching length compared with other groups (Figure 4J), indicating enhanced angiogenic capacity. VEGF is recognized as a key regulator of angiogenesis during tissue repair [36, 40]. The engagement of VEGF with its receptors transduces potent signals via key downstream molecules such as Akt, a kinase that supports endothelial cell function. To further assess the pro‐angiogenic effect of TB@CuPNS, VEGF and Akt mRNA expression in HUVECs was measured using the same qRT‐PCR approach (Figure 4L and Table S2). The results showed that VEGF and Akt transcript levels were significantly elevated in the TB@CuPNS group compared with the other groups, suggesting that TB@CuPNS may enhance angiogenic activity by upregulating VEGF expression and Akt‐related signaling. Together, these findings demonstrate that TB@CuPNS sponges exhibit favorable cytocompatibility and support multiple cellular behaviors required for wound repair, including fibroblast proliferation, fibroblast migration, ECM deposition, keratinocyte viability and migration, and endothelial tube formation. These results provide in vitro evidence for the regenerative potential of TB@CuPNS in chronic wound repair.

### 2.5 In Vitro Antioxidant Activity and Macrophage Immunomodulation

The early inflammatory phase of wound healing is initiated by macrophage‐mediated responses [41]. During this phase, macrophages predominantly adopt a pro‐inflammatory M1 phenotype, facilitating pathogen clearance and phagocytosis of debris [42]. Subsequent polarization toward an anti‐inflammatory M2 phenotype suppresses excessive inflammation and drives tissue remodeling [8]. However, dysregulated elevation of inflammatory mediators after injury often exacerbates tissue damage and impairs regenerative processes [43].

**FIGURE 5:**
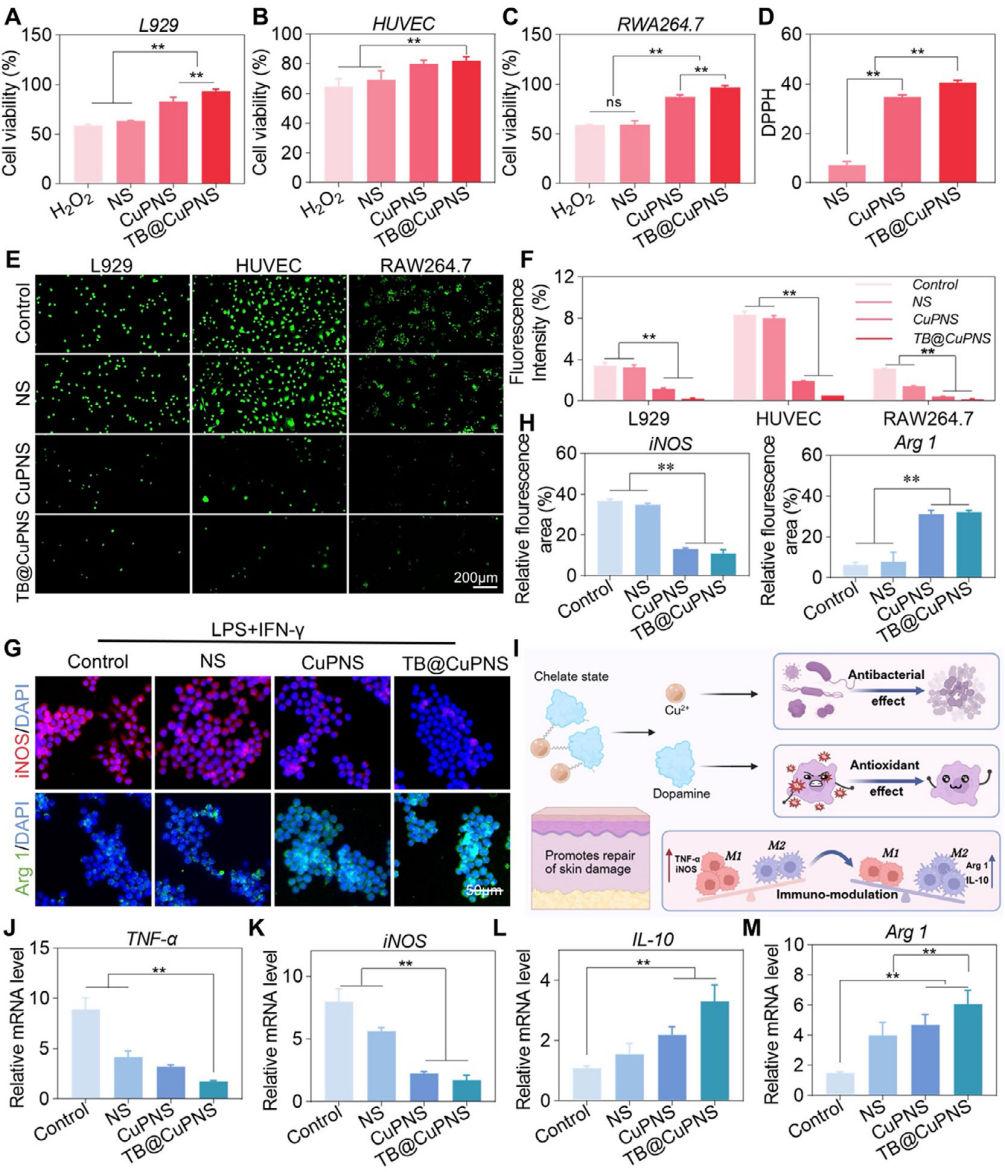
In vitro antioxidant properties and immunomodulatory effects of TB@CuPNS on macrophages. (A‐C) Cell viability of L929 fibroblasts (A), HUVECs (B), and RAW264.7 macrophages (C) after exposure to H_2_O_2_. (D) Total ROS scavenging efficiency of NS, CuPNS, and TB@CuPNS. (E) Fluorescence images of 2',7'‐dichlorofluorescin diacetate (DCFH‐DA) staining for intracellular ROS levels in the three cell lines. (F) Quantification of the ROS‐related fluorescence intensity. (G) Immunofluorescence images of macrophages stained for iNOS and ARG1. (H) Transcript levels of iNOS and ARG1 in RAW264.7 macrophages stimulated with LPS + IFN‐γ. (I) Schematic diagram of the immune regulation mechanism by which TB@CuPNS regulates macrophage polarization and remodels the immune microenvironment through antibacterial, antioxidant effects, and copper ion regulation. (J‐M)Relative mRNA levels of M1 biomarkers (iNOS and TNF‐α) and M2 biomarkers (ARG1 and IL‐10). (A‐D, F, H, J‐M) Statistical analysis was performed using one‐way ANOVA with Tukey's post hoc test. *n* = 3 independent experiments per group. All data in the graphs are presented as mean ± SD; ^**^ *p* < 0.01.

To investigate the cytoprotective and immunoregulatory effects of TB@CuPNS under oxidative stress and inflammatory conditions, in vitro experiments were conducted using L929 fibroblasts, HUVECs, and RAW264.7 macrophages. When exposed to hydrogen peroxide (H_2_O_2_) stimulation, the cell viability of L929, HUVEC, and RAW264.7 cells decreased significantly. Compared with the NS group, the CuPNS and TB@CuPNS groups exhibited better resistance to H_2_O_2_‐induced damage and maintained cell viability. Notably, TB@CuPNS markedly improved cell survival across all three cell types (Figure 5A–C), indicating its potent antioxidant capacity. This effect may be attributed to the ROS‐scavenging activity of the network formed by Cu^2^^+^ and polyphenols, in which PDA contributes antioxidant and immunomodulatory properties as a polyphenol‐derived biomaterial [44]. ROS levels were further evaluated using a total ROS scavenging assay and fluorescence‐based intracellular ROS detection.

TB@CuPNS significantly reduced intracellular ROS levels in all tested cell types, as confirmed by 2′,7′‐dichlorodihydrofluorescein diacetate (DCFH‐DA) probe staining (Figure 5E) and corresponding fluorescence intensity quantification (Figure 5F). These findings were consistent with the total ROS scavenging results (Figure 5D), indicating that TB@CuPNS effectively attenuates excess oxidative stress, a key factor contributing to chronic wound persistence.

The immunomodulatory role of TB@CuPNS was further assessed in RAW264.7 macrophages stimulated with LPS and IFN‐γ. To examine whether ROS scavenging helps restore M1/M2 homeostasis, a series of assays targeting M1 and M2 biomarkers were performed. Among these biomarkers, ARG1 promotes collagen deposition and epithelialization, whereas IL‐10 suppresses pro‐inflammatory cell activities, including activation, migration, and adhesion. Compared with the NS and CuPNS groups, gene expression analysis (Figure 5J–M and Table S2) showed that TB@CuPNS mitigated inflammation by suppressing pro‐inflammatory factors including TNF‐α and iNOS, while elevating anti‐inflammatory mediators such as ARG1 and IL‐10. These gene‐level results were supported by immunofluorescence staining for iNOS and ARG1 (Figure 5G,H), which clearly demonstrated a shift from the M1 phenotype toward the M2 phenotype. A schematic illustration (Figure 5I) summarizes the protective mechanism of TB@CuPNS, highlighting its dual role in reducing oxidative stress and regulating macrophage polarization.

In summary, these results show that TB@CuPNS helped restore macrophage polarization balance under pro‐inflammatory conditions in vitro by scavenging excess ROS. Beyond remodeling immune homeostasis, photothermal TB@CuPNS counteracted oxidative stress damage and reduced pro‐inflammatory cytokine secretion by suppressing macrophage M1 activation. These combined effects help limit chronic wound inflammation and create a supportive microenvironment for wound healing.

### 2.6 Blood Compatibility and In Vivo Hemostatic Performance

**FIGURE 6:**
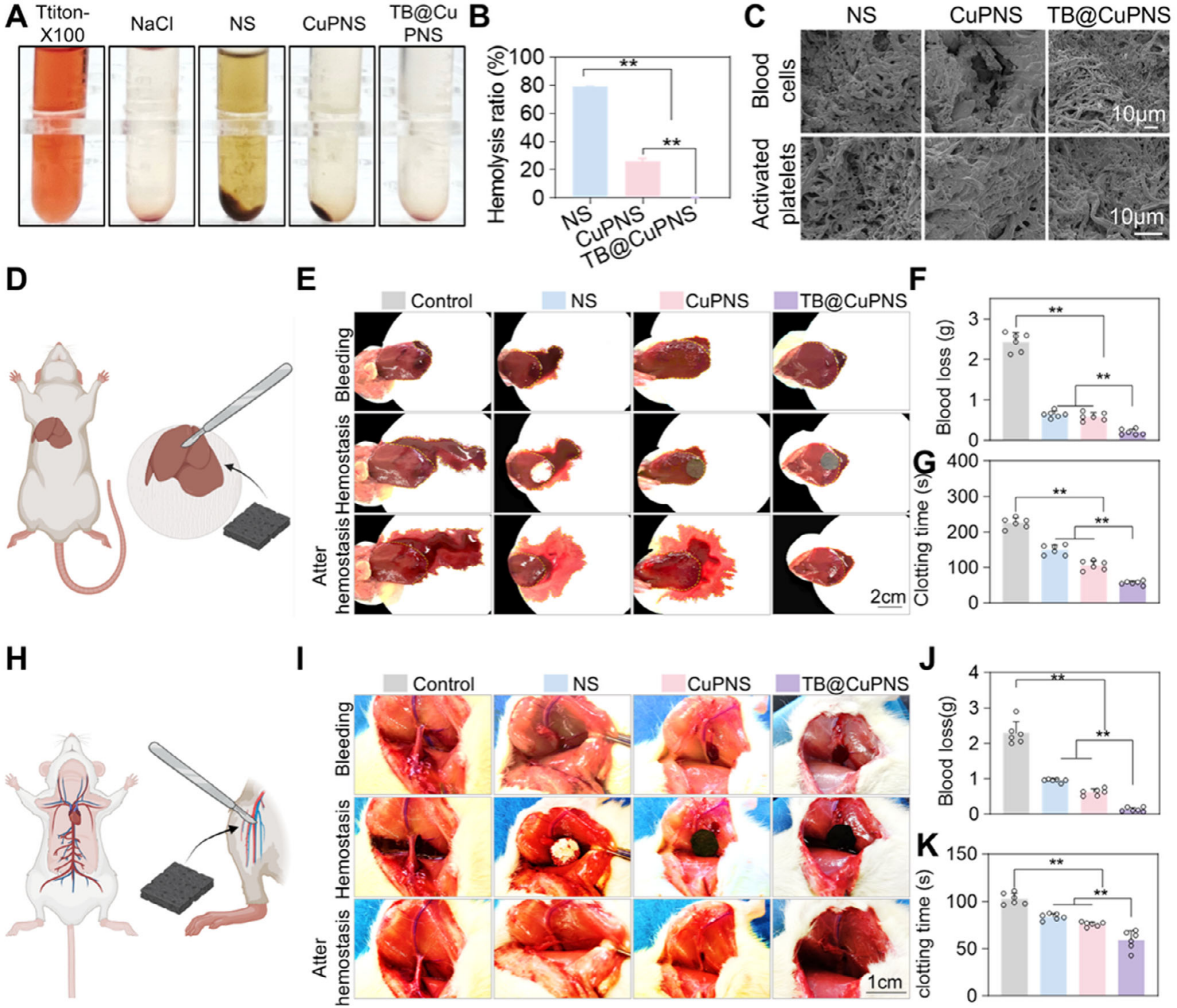
Evaluation of TB@CuPNS for in vivo hemostasis in rat models. (A) Photographs of hemolysis tests for NS, CuPNS, and TB@CuPNS. (B) Quantification of hemolysis rates of NS, CuPNS, and TB@CuPNS. (C) Representative scanning electron microscopy (SEM) images of red blood cells (RBCs) and activated platelet adhesion on different sponges. (D,E) Schematic illustration (D) and representative images (E) of hemostasis in the rat liver incision model. (F,G) Quantification of blood loss (F) and clotting time (G) in the rat liver incision model. (H,I) Schematic illustration and representative images of the rat femoral artery hemorrhage model. (J,K) Quantification of blood loss (J) and clotting time (K) in the rat femoral artery hemorrhage model. (B, F, G, J, K) Statistical analysis was performed using one‐way ANOVA with Tukey's post hoc test. For the hemolysis assay (B), *n* = 3 independent experiments per group; for the rat liver and femoral artery hemostasis experiments (F, G, J, and K), *n* = 6 animals per group. All data in the graphs are presented as mean ± SD; ^**^ *p* < 0.01.

Biocompatibility is a crucial characteristic of hemostatic materials in clinical applications. The blood compatibility and hemostatic ability of TB@CuPNS were assessed through hemolysis tests, cellular adhesion assays, and bleeding control models in rats. A marked hemolytic response was observed in the Triton X‐100 group, as reflected by its bright red appearance in Figure 6A. Conversely, the TB@CuPNS and NaCl groups showed similar hues, indicating minimal hemolysis. Quantitative analysis (Figure 6B) showed that the hemolysis rates of CuPNS and TB@CuPNS decreased to 26.4% and 0.3%, respectively, compared with 79.1% in the NS group. This considerable reduction in RBC lysis confirmed the excellent blood compatibility of TB@CuPNS, which is essential for its potential use as a hemostatic material in clinical settings.

Erythrocytes and platelets mainly contribute to hemostasis and thrombosis through adhesion and aggregation. Red blood cells support clot formation through their physical properties and act in concert with platelets, while platelets drive clot formation through adhesion, aggregation, and cascade amplification. This cooperation rapidly seals the wound and ensures effective bleeding control. To characterize cell adhesion on the material surface, red blood cells and platelets were visualized by SEM (Figure 6C). Compared with the CuPNS and NS groups, where only minimal red blood cells were observed, the TB@CuPNS group demonstrated markedly enhanced and more concentrated erythrocyte aggregation. SEM evaluation of platelet adhesion further showed that TB@CuPNS had higher platelet adhesion than the CuPNS and NS groups. These findings indicate that TB@CuPNS promotes the aggregation of both red blood cells and platelets, further supporting its hemostatic potential.

To evaluate hemostatic efficacy in vivo, rat models of hepatic and femoral artery hemorrhage were established. Total blood loss and hemostasis time were measured in the Control, NS, CuPNS, and TB@CuPNS groups. In the rat liver surface wound model, a 1 cm incision was made on the liver (Figure 6D). Compared with the Control, NS, and CuPNS groups, TB@CuPNS sealed the wound rapidly after bleeding, maintained good tissue adhesion, and markedly reduced blood loss (Figure 6E). Quantitative analysis of the liver model (Figure 6F,G) showed that the TB@CuPNS group demonstrated significantly reduced blood loss (0.21 ± 0.07 g) and shortened hemostasis time (56.53 ± 5.42 s), compared with NS (0.64 ± 0.08 g, 149.29 ± 13.88 s) and CuPNS (0.59 ± 0.10 g, 106.01 ± 12.68 s).

In the rat femoral artery model, hemorrhage was induced by puncture using a 10 mL syringe needle (Figure 6H). Hemostatic performance was evaluated in the Control, NS, CuPNS, and TB@CuPNS groups, with representative images shown in Figure 6I. The Control and NS groups exhibited severe and prolonged bleeding, with diffuse blood accumulation around the wound. In contrast, the CuPNS group showed partial bleeding control, whereas the TB@CuPNS group rapidly reduced bleeding with negligible blood leakage after application. This visual evidence was consistent with the quantitative results, showing that TB@CuPNS provided the most effective bleeding control, with blood loss of 0.13 ± 0.06 g and hemostasis time of 59.05 ± 9.93 s, outperforming the NS group (0.95 ± 0.04 g, 83.74 ± 3.33 s) and CuPNS group (0.62 ± 0.10 g, 75.92 ± 2.30 s) (Figure 6J,K).

Overall, these findings confirm that TB@CuPNS exhibits excellent blood compatibility and effective hemostatic performance under both low‐ and high‐pressure bleeding conditions, supporting its translational potential as a hemostatic material for wound repair.

### 2.7 In Vivo Antibacterial Efficacy and *S. aureus*‐Infected Diabetic Wound Healing Repair

**FIGURE 7:**
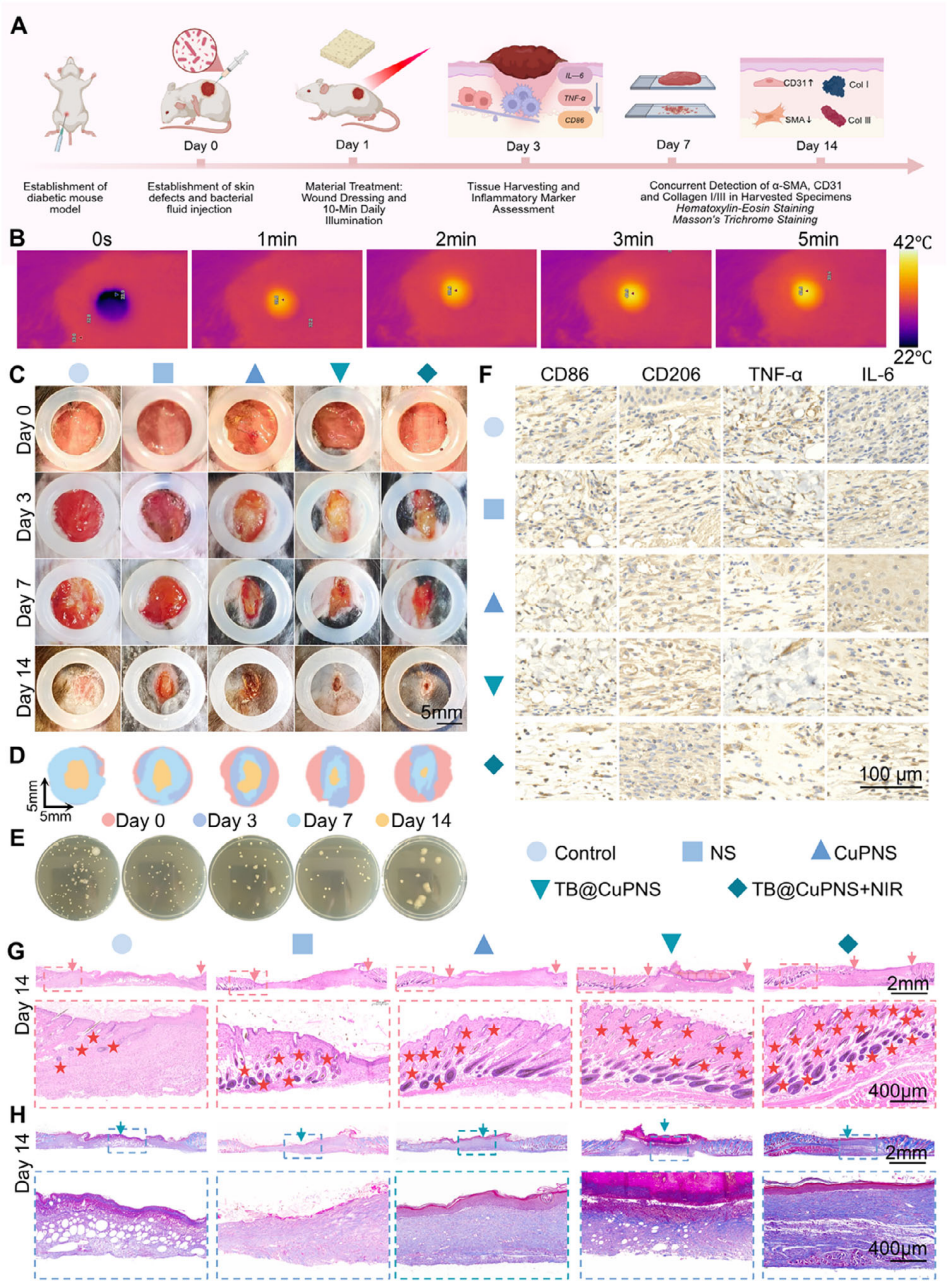
In vivo repair of *S. aureus*‐infected diabetic wounds. (A) Schematic illustration of the experimental process for establishing the *S. aureus*‐infected diabetic wound model. (B) Representative infrared thermographic images of wound sites during 808 nm NIR irradiation. (C) Representative photographs showing wound healing progression in different groups on days 0, 3, 7, and 14. (D) Quantified maps of the wound healing process. (E) Representative bacterial colony‐forming unit (CFU) cultures from wound tissues in different treatment groups after 2 days of treatment. (F) Immunohistochemical staining images for CD86, CD206, TNF‐α, and IL‐6 in wound tissues on day 3 after treatment. (G) H&E staining images of wounds from different groups on day 14 (red arrows: granulation tissue gap; red stars: regenerated hair follicles). (H) Masson's trichrome staining images of wounds from different groups on day 14 (blue arrows: wound center).

Building on its mild photothermal antibacterial activity and in vitro ability to regulate fibroblast proliferation and migration, keratinocyte viability and migration, collagen deposition, HUVEC proliferation, and angiogenesis, TB@CuPNS was further evaluated in an *S. aureus*‐infected diabetic full‐thickness skin wound model (Figure 7A). All animal procedures were approved by the Animal Ethics Committee of Peking University People's Hospital (approval No. 2023PHE088).

Diabetes was induced in mice by intraperitoneal injection of streptozotocin (STZ) [45], and only mice with persistently elevated blood glucose levels above 16.7 mmol/L were used for wound experiments. After a 10‐mm full‐thickness dorsal skin wound was created, 3.5 × 10^6^ CFU of *S. aureus* was inoculated onto the wound bed to establish an infected diabetic wound model. To evaluate the in vivo photothermal performance of TB@CuPNS sponges, diabetic mice were exposed to 808 nm NIR light at the wound site, and temperature changes were continuously monitored using an infrared thermal imaging camera (Figure 7B). The wound surface temperature rapidly increased and stabilized at approximately 45°C within a few minutes, consistent with the mild photothermal range selected for antibacterial treatment. These results confirm that TB@CuPNS can serve as a controllable in vivo photothermal agent at the infected wound site.

The healing process of infected full‐thickness diabetic wounds was monitored over 14 days, with representative images acquired on days 0, 3, 7, and 14 (Figure 7C). To ensure transparent wound closure quantification, representative wound size measurement markers used for calculating wound area are provided in Figure S9. Wound closure rates on days 3, 7, and 14 are shown in Figure S10. Although all groups followed a progressive healing trend, clear differences in healing efficiency were observed. The TB@CuPNS + NIR group exhibited markedly accelerated wound contraction at all time points compared with the other groups.

At 7 days post‐surgery, wounds in the Control and NS groups remained moist, with a bright red appearance and limited scab formation. In contrast, wounds treated with CuPNS, TB@CuPNS, and TB@CuPNS + NIR appeared drier and showed visible scab formation, which may be attributed to the water absorption and antibacterial properties of these scaffolds. Under 808 nm NIR irradiation, TB@CuPNS combined mild photothermal activation with sponge‐mediated exudate management, promoting Cu^2^^+^ release, reducing excessive exudate accumulation, enhancing antibacterial activity, and helping maintain a favorable wound microenvironment. By day 14, the wound area in all groups was significantly reduced compared to day 7. Quantitative analysis (Figure 7D and Figure S10) showed that the wound healing rates on day 14 were 68.80% (Control), 78.33% (NS), 89.74% (CuPNS), and 92.53% (TB@CuPNS). Remarkably, the TB@CuPNS + NIR group achieved a significantly higher healing rate of 97.55%, demonstrating superior wound healing efficacy compared to the TB@CuPNS and CuPNS groups. Because this model contained a defined *S. aureus* bacterial burden, bacterial colony culture of wound tissues was further performed to directly assess the in vivo anti‐infection efficacy of TB@CuPNS (Figure 7E). The TB@CuPNS + NIR group showed a substantial reduction in wound bacterial load, with an antibacterial rate of 92%, which was higher than those of the TB@CuPNS (78%) and CuPNS (64.67%) groups (Figure S11). These data demonstrate that TB@CuPNS + NIR effectively suppresses bacterial infection in the *S. aureus*‐infected diabetic wound model through the synergistic effects of Cu^2^^+^‐mediated antibacterial activity and mild photothermal therapy.

The inflammatory microenvironment acts as a key obstacle to tissue regeneration. TNF‐α and IL‐6, two main pro‐inflammatory cytokines, contribute to this barrier, and their elevated levels are hallmarks of acute and chronic inflammation. IHC staining for IL‐6 and TNF‐α on day 3 showed significantly lower signals in the TB@CuPNS + NIR group compared to other groups, indicating effective suppression of inflammatory responses (Figure 7F and Figure S12). Macrophages play an essential role in clearing necrotic cells, regulating immune responses, and maintaining tissue homeostasis [46]. M1 macrophages primarily initiate and sustain pro‐inflammatory responses [47]. However, sustained M1 activation can lead to chronic inflammation and tissue damage. In contrast, M2 macrophages are primarily involved in anti‐inflammatory modulation, tissue repair, and fibrosis [48], maintaining immune homeostasis by inhibiting excessive immune responses and preventing tissue damage caused by hyperinflammation. IHC analysis of CD86 and CD206 on day 3 further showed reduced CD86‐positive staining and increased CD206‐positive staining in the TB@CuPNS + NIR group compared to others, highlighting the immunomodulatory effect of TB@CuPNS under mild photothermal activation.

To better understand tissue repair after treatment, histological analyses were performed using H&E and Masson's trichrome staining. Representative H&E and Masson's trichrome images on day 14 are shown in Figure 7G,H, while the corresponding images on day 7 are provided in Figures S13 and S14. On day 7, the TB@CuPNS + NIR treatment group exhibited epidermal tissue regeneration, thicker re‐epithelialized tissue, newly formed hair follicles, reduced inflammatory cell infiltration, and a scab covering the wound compared with the other groups. On day 14, the TB@CuPNS + NIR group exhibited the most advanced tissue remodeling, with regular epithelial stratification, dense collagen deposition, and more mature dermal structures. In contrast, the other groups showed incomplete epidermal regeneration, edema, and ongoing persistent inflammation. Quantitative analysis of histological sections further confirmed that TB@CuPNS + NIR reduced scar width and increased newly formed granulation tissue thickness at both day 7 and day 14, with the most pronounced differences observed at day 14 (Figures S15 and S16). On day 14, compared with the Control group (scar width: 8.09 ± 6.49 mm, granulation tissue thickness: 591.83 ± 36.62 µm), NS group (6.97 ± 0.47 mm, 668.43 ± 22.72 µm), CuPNS group (6.07 ± 0.19 mm, 775.53 ± 36.12 µm), and TB@CuPNS group (5.04 ± 0.15 mm, 902.63 ± 68.04 µm), the TB@CuPNS + NIR group exhibited the shortest scar width (3.76 ± 0.69 mm) and the thickest newly formed granulation tissue (1121.97 ± 96.08 µm). These histological findings, together with the enhanced collagen organization observed by Masson's trichrome staining, indicate accelerated ECM remodeling and more mature tissue repair in the TB@CuPNS + NIR group.

The local tolerance and systemic safety of TB@CuPNS under the wound treatment conditions were further assessed through gross wound observations, wound tissue histology, and major organ histology. During the 14 days of treatment, gross observation showed no obvious additional skin edema, necrosis around the wound, or abnormal tissue damage in the TB@CuPNS + NIR group. Histological analysis further revealed reduced inflammatory infiltration, more continuous epidermal regeneration, and improved dermal organization in the TB@CuPNS + NIR group, indicating that mild photothermal treatment did not aggravate local tissue injury in the infected diabetic wound model. In addition, H&E staining of major organs, including the heart, liver, spleen, lung, and kidney, showed no obvious pathological abnormalities after treatment, supporting the favorable in vivo safety profile of TB@CuPNS (Figure S19).

Together, TB@CuPNS sponges combined with NIR irradiation promoted effective healing of infected diabetic wounds through a coordinated mechanism involving bacterial infection control, excessive inflammation reduction, and activation of regenerative processes. These findings highlight the potential of TB@CuPNS as an advanced multifunctional platform for infected chronic wound repair.

### 2.8 Immunohistochemical Evaluation of Neovascularization and Collagen Remodeling

**FIGURE 8:**
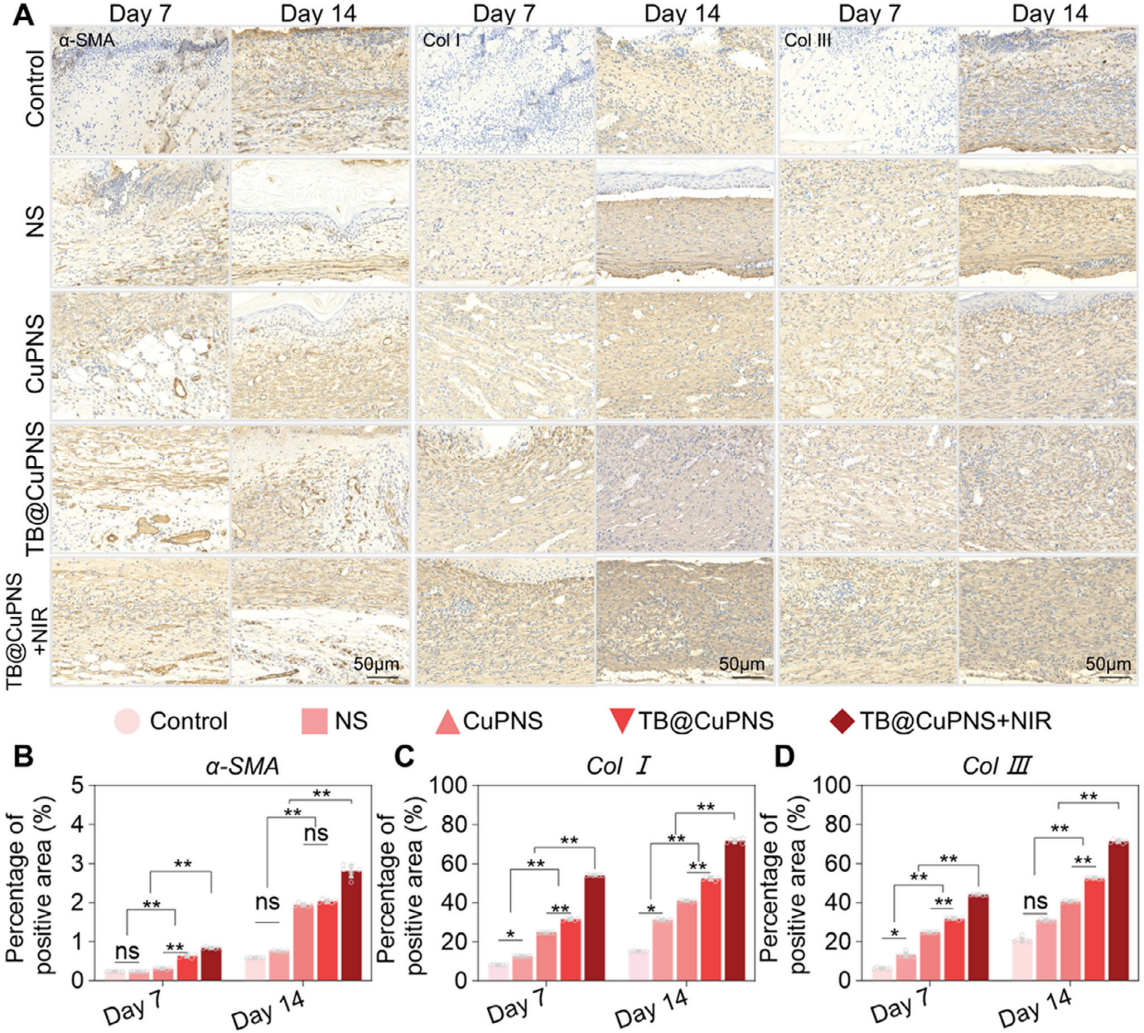
Immunohistochemical (IHC) evaluation of neovascularization and collagen remodeling during infected diabetic wound healing. (A) Representative IHC staining images of α‐SMA, Col I, and Col III in wound tissues from the Control, NS, CuPNS, TB@CuPNS, and TB@CuPNS + NIR groups on days 7 and 14. (B‐D) Quantification of positive staining area for α‐SMA (B), Col I (C), and Col III (D) on days 7 and 14. (B‐D) Statistical analysis was performed using one‐way ANOVA with Tukey's post hoc test. *n* = 6 animals per group. All data in the graphs are presented as mean ± SD; ^*^ *p* < 0.05, ^**^ *p* < 0.01.

Collagen remodeling at the wound bed and neovessel formation are common signs of skin regeneration and remodeling after injury [49]. To further assess the role of TB@CuPNS in tissue regeneration beyond macroscopic wound closure, IHC staining was performed for α‐SMA, Col I, and Col III in wound tissues on days 7 and 14 (Figure 8A). CD31 IHC staining was also performed to evaluate neovascularization, with representative images and quantification provided in Figures S17 and S18. Together, these markers reflect key repair processes, including fibroblast activation, myofibroblast differentiation, angiogenesis, and ECM reorganization.

α‐SMA‐positive staining reflects myofibroblast‐associated tissue contraction and remodeling, whereas CD31‐positive staining identifies endothelial structures associated with neovascularization during wound repair. On days 7 and 14, α‐SMA staining was stronger and more organized in the TB@CuPNS + NIR group than in the other groups (Figure 8A,B), indicating enhanced myofibroblast activation and tissue remodeling. CD31 IHC staining further revealed dynamic vascular remodeling during wound repair (Figures S17 and S18). On day 7, the TB@CuPNS + NIR group exhibited increased CD31‐positive vascular structures, suggesting active neovascularization during the early healing phase. By day 14, CD31 expression in the TB@CuPNS + NIR group was not further elevated compared with some other treatment groups, which may reflect the transition from active angiogenesis toward vascular maturation and tissue remodeling as wound healing progressed.

Beyond vascular remodeling, collagen deposition is a crucial factor in skin tissue reconstruction. Collagen I and III, the major structural components of the dermal extracellular matrix (ECM), play a crucial role in wound healing, as their ordered deposition and subsequent remodeling enhance tissue tensile strength and improve healing outcomes. IHC staining showed that Col I‐ and Col III‐positive areas increased during healing, with the TB@CuPNS + NIR group forming the most abundant and well‐organized matrix (Figure 8A). Quantitative analysis further confirmed that the TB@CuPNS + NIR group exhibited significantly higher Col I‐ and Col III‐positive staining areas than the other groups (Figure 8C,D), suggesting accelerated ECM deposition and collagen remodeling.

Overall, these results show that TB@CuPNS, especially under NIR activation, promotes infected diabetic wound healing by enhancing angiogenesis and increasing collagen synthesis. Together with the antibacterial and immunomodulatory results described above, these findings indicate that TB@CuPNS creates a wound microenvironment favorable for tissue repair through coordinated infection control, inflammation regulation, mild photothermal activation, and Cu^2^^+^‐associated bioactivity.

### 2.9 Transcriptomic Analysis and qRT‐PCR Validation of Wound‐Repair Mechanisms

**FIGURE 9:**
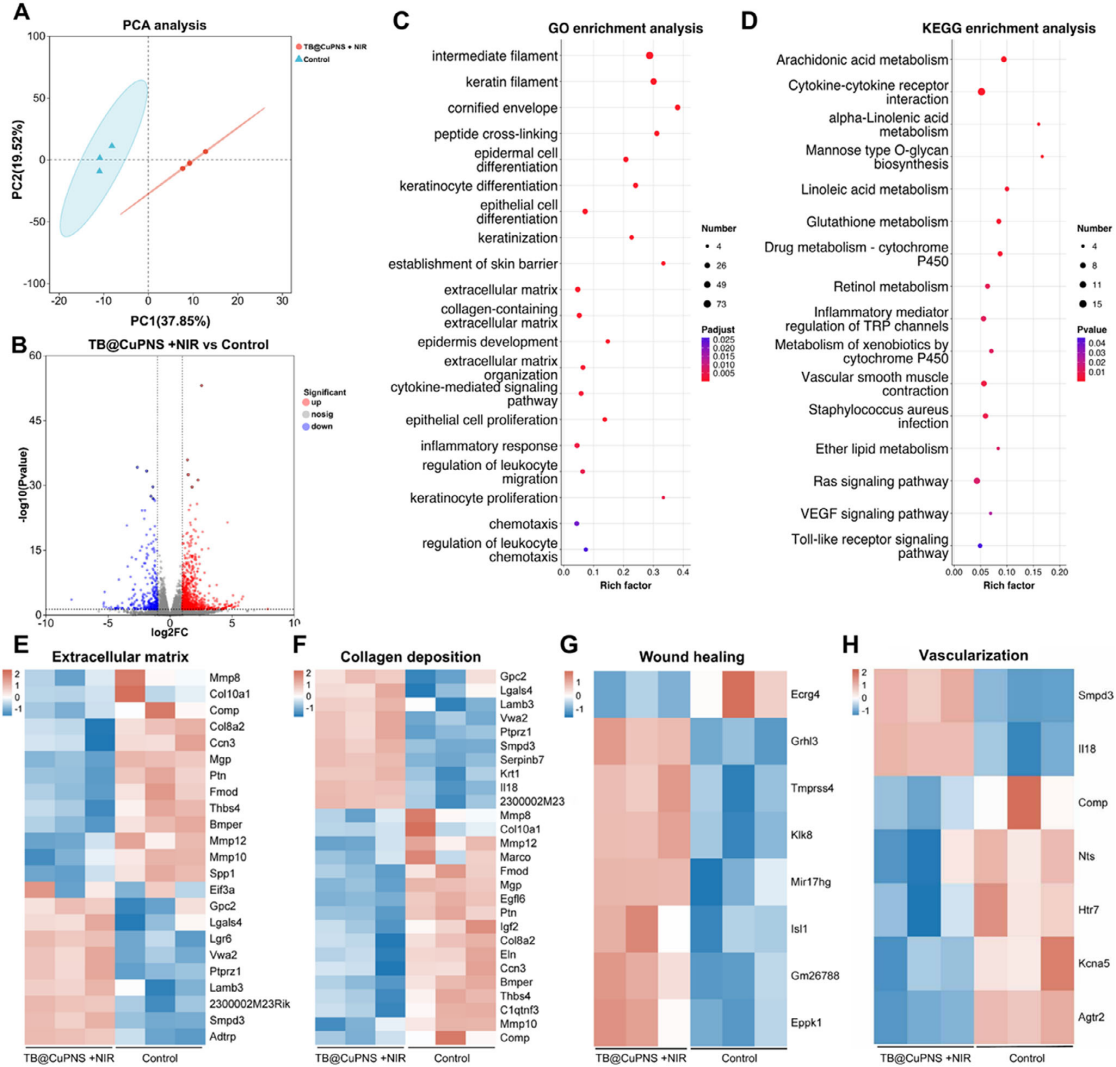
Transcriptomic analysis of wound‐repair mechanisms after TB@CuPNS + NIR treatment.  (A) Principal component analysis (PCA) showing transcriptional separation between the Control and TB@CuPNS + NIR groups in wound tissues collected on day 7. (B) Volcano plot showing upregulated and downregulated differentially expressed genes (DEGs) in the TB@CuPNS + NIR group compared with the Control group. (Red dots: upregulated DEGs, blue dots: downregulated DEGs, and gray dots: nonsignificant genes). (C) Gene Ontology (GO) enrichment analysis of DEGs. (D) Kyoto Encyclopedia of Genes and Genomes (KEGG) pathway enrichment analysis of DEGs. (E‐H) Heatmaps illustrating representative DEGs associated with extracellular matrix remodeling (E), collagen deposition (F), wound healing (G), and vascularization (H). DEGs were filtered using the thresholds of |log_2_(fold change)| ≥ 1 and *p* < 0.05. For RNA‐seq analysis, *n* = 3 per group.

To further identify the mechanism by which NIR‐activated TB@CuPNS enhances infected diabetic wound healing, bulk RNA sequencing was performed using wound tissues collected from the Control and TB@CuPNS + NIR groups on day 7. Principal component analysis (PCA) showed a clear separation between the two groups (PC1: 37.85%; PC2: 19.52%), indicating distinct transcriptional profiles after NIR‐activated TB@CuPNS treatment (Figure 9A). Differential expression analysis further identified 1149 differentially expressed genes (DEGs), including 848 upregulated and 301 downregulated genes in the TB@CuPNS + NIR group compared with the Control group (Figure 9B).

GO enrichment analysis revealed biological processes related to tissue repair, involving epithelial regeneration, restoration of the skin barrier, ECM remodeling, inflammatory regulation, chemotaxis, and leukocyte migration (Figure 9C). These results suggest that TB@CuPNS + NIR treatment promotes coordinated epithelial repair, matrix reconstruction, and immune cell regulation during wound healing (Figure 9D). KEGG enrichment analysis further identified pathways associated with inflammatory regulation, oxidative and metabolic remodeling, angiogenesis, host defense, and immune regulation. These pathways are closely associated with coordinated antibacterial, antioxidant, angiogenic, and immunoregulatory effects in the wound microenvironment.

Heatmaps of representative DEGs further supported these enrichment results (Figure 9E–H). Genes associated with epithelial repair and barrier reconstruction, including Grhl3, Tmprss4, Klk8, Isl1, and Eppk1, were increased after TB@CuPNS + NIR treatment. In contrast, several genes involved in inflammation and matrix degradation, including Mmp8, Mmp10, Mmp12, Spp1, Ccl5, and Cxcl10, were reduced, suggesting attenuated inflammatory matrix turnover after TB@CuPNS + NIR treatment. Meanwhile, genes associated with basement membrane reconstruction and epithelial matrix interactions, such as Lamb3, Vwa2, Ptprz1, Gpc2, and Lgals4, showed expression changes consistent with improved ECM remodeling. Together, these transcriptional patterns support a shift from inflammatory tissue degradation toward epithelial regeneration and constructive matrix remodeling.

To validate representative RNA‐seq findings, eight genes were selected for qRT‐PCR analysis. The primer sequences used for qRT‐PCR validation are listed in Table S3. Krt14, Krt16, Grhl3, and Lamb3 were selected to represent keratinocyte activation, epithelial differentiation, barrier repair, and basement‐membrane reconstruction, whereas Mmp12, Marco, Ccl5, and Cxcl10 were selected to represent macrophage‐associated inflammation and chemokine signaling. qRT‐PCR validation confirmed the RNA‐seq trends, with Krt14, Krt16, Grhl3, and Lamb3 upregulated and Mmp12, Marco, Ccl5, and Cxcl10 downregulated in the TB@CuPNS + NIR group (Figures S20 and S21). These results provide independent molecular validation that TB@CuPNS + NIR treatment promotes epidermal regeneration and matrix reconstruction while attenuating excessive inflammatory and chemokine responses.

In summary, TB@CuPNS + NIR treatment induces broad transcriptional reprogramming in infected diabetic wound tissues and coordinately regulates epithelial differentiation, ECM remodeling, angiogenesis, and inflammation resolution. This transcriptomic evidence provides a mechanistic basis for the superior therapeutic performance of TB@CuPNS under NIR irradiation in infected chronic wound healing.

## 3 Conclusions

Here, we developed a photothermal 3D nanofiber sponge (TB@CuPNS) assembled through metal‐polyphenol coordination, integrating mild photothermal therapy (PTT), chemodynamic therapy (CDT), and immunomodulatory functions for chronic wound repair. Under near‐infrared (NIR) irradiation, TB@CuPNS generated mild localized hyperthermia (around 45°C), which worked together with copper ion (Cu^2^^+^) release to kill bacteria while reducing the risk of thermal injury to surrounding tissues. Importantly, Cu^2^^+^ release from TB@CuPNS responds to pH and redox signals, enhancing antibacterial power by promoting the specific generation of reactive oxygen species (ROS) and the targeted depletion of glutathione (GSH), thereby targeting both planktonic bacteria and bacterial biofilms. In addition to its antibacterial properties, TB@CuPNS exhibited effective hemostatic performance in rat liver and femoral artery hemorrhage models, which was attributed to its porous architecture and thrombin functionalization. The sponge also efficiently managed wound exudate, maintaining an optimal moist environment while preventing excessive fluid accumulation. Comprehensive in vitro and in vivo studies confirmed the excellent biocompatibility of TB@CuPNS and its ability to promote fibroblast proliferation, collagen matrix deposition, and neovascularization, processes critical for functional skin tissue regeneration. Further RNA‐seq of wound tissue samples from diabetic mice demonstrated that NIR‐activated TB@CuPNS modulates epithelial cell differentiation, extracellular matrix remodeling, neovascularization, and inflammation resolution through pivotal signaling and metabolic pathways.

In conclusion, TB@CuPNS integrates rapid hemostasis, broad‐spectrum antibacterial activity, wound exudate regulation, and immunomodulatory functions within a single therapeutic platform. Its capacity to respond to the pathological wound microenvironment and coordinate multiple stages of tissue repair supports its potential as an advanced dressing for chronic infected wounds.

## 4 Experimental Section

Materials, experimental methods, preparation and characterization of the TB@CuPNS sponge, in vitro antibacterial assays, cytocompatibility and cell behavior studies, antioxidant and immunomodulatory evaluations, hemostatic assays, in vivo infected diabetic wound‐healing experiments, histological and immunohistochemical analyses, RNA sequencing, and qRT‐PCR validation are provided in the Supporting Information.

### 4.1 Animal Experiment

All in vivo experimental procedures were conducted in compliance with relevant ethical standards and were approved by the Animal Ethics Committee of Peking University People's Hospital (approval No. 2023PHE088).

### 4.2 Statistical Analysis

All numerical data are presented as mean ± standard deviation (SD). Statistical analyses were performed using an unpaired Student's t‐test for two‐group comparisons or one‐way analysis of variance (ANOVA) followed by Tukey's post hoc test for multiple‐group comparisons. Differences were considered statistically significant at *p* < 0.05. Significance levels are denoted as ^*^ *p* < 0.05, ^**^ *p* < 0.01, ^***^ *p* < 0.001, and ^****^ *p* < 0.0001.

## Author Contributions

Z. Z. conceived the overall experimental design and oversaw the coordination of the research project. Z. Z., F. Q., and X. Z. collated, integrated, and refined the experimental data and analytical results. Z. Z. and F. Q. performed and analyzed the in vivo animal experiments, while Z. Z. and X. S. conducted and interpreted the RNA sequencing analyses. Z. Z., F. Q., and X. S. drafted the initial version of the manuscript and prepared the original research draft. J. Z., M. C., B. L., M. S., and Y. B. contributed to the critical revision and review of the final manuscript. Y. W., M. L., and D. Z. provided supervision and guidance during the final revision and editing of the manuscript.

## Conflicts of Interest

The authors declare no conflicts of interest.

## Supporting information




**Supporting File 1**: advs77534‐sup‐0001‐SuppMat.docx.

**Supporting File 2**: advs77534‐sup‐0002‐S1.mp4.

**Supporting File 2**: advs77534‐sup‐0003‐S2.mp4.

**Supporting File 4**: advs77534‐sup‐0003‐S3.mp4.

## Data Availability

Research data are not shared.
