# Supplementary material for: A Metal‐Polyphenol Self‐Assembled Coated 3D Sponge With Mild Photothermal Antibacterial, Exudate‐Controlling, and Immunomodulatory Functions for Hemostasis and Chronic Wound Healing
Source: Adv Sci (Weinh). 2026 Sep 8:e77534. Online ahead of print. doi: 10.1002/advs.77534 (PMC13554499; doi:10.1002/advs.77534)
Supplement: Supplementary file 1 [file ADVS-9999-e77534-s003.docx]

**Supporting Information**

**A Metal-Polyphenol Self-Assembled Coated 3D Sponge with Mild Photothermal Antibacterial, Exudate-Controlling, and Immunomodulatory Functions for Hemostasis and Chronic Wound Healing**

Ziwei Zhang*^†^*, FengQin*^†^*, Xiaomeng Zhang*^†^*, Xueqin Shuang*^†^*, Jiahua Zhou, Ming Cheng , Baicheng Lu, Mingyue Song, Yuntian Bai , Dianying Zhang*, Ming Li *,Yanhua Wang*

Z. Zhang, F.Qin, X. Zhang, D. Zhang and Y.Wang

Department of Orthopedics and Trauma, Peking University People's Hospital, Beijing100044, China;Key Laboratory of Trauma and Neural Regeneration, Ministry of Education, Peking University, Beijing, 100044, China;National Center for Trauma Medicine, Beijing, 100044, China.

E-mail: [wangyanhua04119@pkuph.edu.cn](mailto:wangyanhua04119@pkuph.edu.cn) and [zhangdianying@bjmu.edu.cn](mailto:zhangdianying@bjmu.edu.cn)

X. Shuang, J. Zhou, M. Cheng , B. Lu, M.Song, Y. Bai and M. Li

Key Laboratory of Trauma and Neural Regeneration, Ministry of Education, Peking University, Beijing, 100044, China;National Center for Trauma Medicine, Beijing, 100044, China;Trauma Medicine Center, Peking University People's Hospital,Beijing, 100044, China.

E-mail: [liming_ort@bjmu.edu.cn](mailto:liming_ort@bjmu.edu.cn)

^#^ Ziwei Zhang, FengQin, Xiaomeng Zhang, Xueqin Shuang contributed equally to this work.

*Correspondence: [wangyanhua04119@pkuph.edu.cn](mailto:wangyanhua04119@pkuph.edu.cn), [liming_ort@bjmu.edu.cn](mailto:liming_ort@bjmu.edu.cn)

and [zhangdianying@bjmu.edu.cn](mailto:zhangdianying@bjmu.edu.cn)

**1. Materials and experiments**

All the experimental procedures were reviewed and approved by the Animal Ethics Committee of Peking University People’s Hospital.

**1.1** **Preparation of the TB@CuPNS sponge**

1.1.1 Preparation of NF fiber scaffold

PU (0.7 g, Sigma-Aldrich, St Louis, MO, USA) and gelatin (0.3 g, Shanghai Aladdin Biochemical Technology Co., LTD., Shanghai, China) were dissolved in 8 mL acetic acid. The mixture was homogenized uniformly and transferred into a 10 mL syringe fitted with a 20-gauge needle for electrospinning. Set the electrospinning parameters as follows: voltage 18.4 kV/-4.2 kV; flow rate 0.15mm/min; roller speed 55rpm; and working distance 18cm. After electrospinning, 0.2 mm-thick NF fiber was prepared and subjected to vacuum drying at ambient temperature overnight to remove any remaining solvents.

1.1.2 Preparation of nanofiber sponge (NS)

The as-prepared electrospun film was sectioned into 0.5 × 0.5 cm squares, after which 1 g of the sample was immersed in 100 mL of tert-butyl alcohol. Stir the mixture at 10,000 rpm for 20 min to achieve uniform dispersion of the fibers. The well-dispersed fiber suspension was transferred to 24-well plates and lyophilized for 24 h. Fix and cross-link with a 2.5% glutaraldehyde solution for 12 h then immerse in a 5% glycine solution for 48 h. Finally, the samples were lyophilized again for an additional 24 h to obtain the 3D electrospun nanofiber sponge (NS).

1.1.3 Preparation of CuPNS nanofiber sponge

A dopamine solution with a concentration of 2 mg/mL was prepared by dissolving dopamine in 10 mM Tris‑HCl (pH = 8.5). The NS was immersed in this dopamine solution in the dark for 12 h, rinsed three times with deionized water, and freeze-dried to obtain the PNS (PDA-coated NS). For the preparation of CuPNS, CuSO₄·5H₂O was added to the solution to achieve final concentrations of 0.1, 0.5, 1.0, and 1.5 mg/mL. The NS was then immersed in the above Cu²⁺-dopamine solution and treated identically to the PNS preparation process to obtain CuPNS with different Cu²⁺ chelation concentrations.

1.1.4 Preparation of TB@CuPNS nanofiber sponge

A thrombin (TB) solution with a concentration of 1 μg/mL was prepared. A 2 mL aliquot of this TB solution was added to separate NS and CuPNS samples, which were then thoroughly infiltrated via ultrasound treatment for 10 min. Afterward, the samples were soaked at room temperature for 12 h to ensure adequate TB adsorption. Finally, the samples were retrieved and freeze-dried to obtain TB@PNS and TB@CuPNS composites, respectively.

**1.2 Characterization of the TB@CuPNS sponge**

1.2.1 Physical morphology

Digital photographs were used to document the appearance and topography of the sponges at each stage of preparation. Scanning electron microscopy (SEM, JSM-7900F, JEOL, Tokyo, Japan) was employed to observe the physical morphology of both 2D nanofiber membranes and 3D nanofiber sponges. Transmission electron microscopy (TEM) analysis was performed to examine microstructure and elemental distribution of TB@CuPNS. Additionally, Fourier-transform infrared spectroscopy (FTIR) and X-ray photoelectron spectroscopy (XPS) were used to analyze the chemical composition and elemental states of the prepared samples.

1.2.2 Porosity

The porosity of the prepared sponges was determined using the liquid-displacement method. Briefly, each dry sponge was weighed and immersed in ethanol in a graduated cylinder for 5 min. After complete wetting, the wet weight was recorded. Porosity was calculated using the following equation: porosity (%) = (Wf - Wi) / (ρ × Wi) × 100%, where Wf is the wet weight of the sponge, Wi is the initial dry weight, and ρ is the density of ethanol.

1.2.3 Absorption property

To evaluate the water absorption capacity of the 3D nanofiber sponges, samples were immersed in deionized water for specific time intervals (0, 0.5, 1, 5, 10, and 30 min), and their absorption ratios were recorded accordingly. Each sample was fully submerged in deionized water; after soaking for the designated time, any excess surface liquid was gently blotted away, and the sample was weighed immediately. The absorption ratio (%) was calculated as  (M1−M0) / M0 × 100, where M0 and M1 represent the dry weight and the soaked wet weight of the 3D nanofiber sponge, respectively.

1.2.4 Mechanical property

The mechanical properties of the 3D nanofiber sponges were tested using a mechanical testing device (Model 5848, Instron, Norwood, MA, USA). Cyclic compression tests were performed on the 3D nanofiber sponge samples, involving 20 loading-unloading fatigue cycles at high compressive strains (ε) of 30%, 50%, and 60%.

**1.3 Photothermal performance of the TB@CuPNS**

To evaluate the photothermal properties of the sponges, NS and CuPNS sponges was first measured in the dry state. The samples were exposed to an 808 nm NIR at a range of power densities (0.05, 0.1, 0.3, 0.5, and 0.7 W·cm⁻²), and their temperature changes were continuously recorded for 3 min. Next, to simulate the physiological microenvironment of chronic wounds, the sponges were placed in PBS, and their temperature profiles were recorded again during 10 minutes of continuous laser irradiation. To assess the photothermal stability of CuPNS, the sponge was evaluated in a wet state (immersed in PBS) over four consecutive ON/OFF cycles. Each cycle included 10 minutes of laser irradiation followed by a natural cooling period to room temperature. Throughout all procedures, real-time temperature changes were monitored and documented using an infrared thermal imaging camera (E8, FLIR, USA).

To assess the sponge's ability to manage wound exudate, a simulated wound exudate evaporation experiment was performed. A salt solution resembling human wound exudate was prepared, containing 0.8298% (w/v) NaCl and 0.0368% (w/v) CaCl₂. Pre-weighed dry sponge samples were fully immersed in the simulated exudate until saturated. After carefully blotting away excess surface liquid, the initial weight of each saturated sponge was recorded. The samples were then divided into two groups: the control group was kept at room temperature, while the experimental group was exposed to continuous NIR laser irradiation (808 nm, 0.7 W·cm⁻²). The weight of the sponges in both groups was measured at specific time points over a 6 h period. The evaporation rate was calculated using the formula: Evaporation Rate (%) = (Initial wet weight - Wet weight at time t) / (Initial wet weight - Dry weight) × 100%, where t indicates the designated time intervals.

**1.4 Cu ions release behavior of** **TB@CuPNS**

Two experimental conditions were established to examine the Cu²⁺ release behavior of TB@CuPNS, with and without NIR (808 nm, 0.7 W·cm⁻²). Briefly, 1 mg of TB@CuPNS sponge samples were immersed in 2 mL of ultrapure water, and the obtained suspensions were either irradiated with the NIR laser or kept unirradiated. At predetermined time points, 1 mL of supernatant was collected and diluted with 5 mL of ultrapure water, then Cu²⁺ concentration was measured in the diluted solution using inductively coupled plasma optical emission spectroscopy (ICP-OES).

To assess the pH-responsiveness of Cu²⁺ release, TB@CuPNS sponges were separately immersed in PBS buffers at pH 7.4 and pH 5.5, the latter simulating the microenvironment of infected chronic wounds. To assess ROS responsiveness, TB@CuPNS sponges were immersed in PBS and H₂O₂ separately, with the H₂O₂ solution mimicking the microenvironment of infected wounds.

**1.****5 Antibacterial property**

1.5.1 In vitro antibacterial test of the TB@CuPNS

Before the antibacterial test, NS, CuPNS, and TB@CuPNS sponge samples were sterilized in 75% ethanol for 2 h, rinsed three times with sterile PBS to eliminate residual ethanol, and then placed in a 24-well plate. Each well was supplemented with 600 μL of *S. aureus* suspension (2.2 × 10⁸ CFU/mL) and 1400 μL of sterile PBS, followed by incubation at 37 °C for 2 h. Afterward, the TB@CuPNS group was subjected to an 808 nm NIR laser (0.7 W·cm⁻²) for 10 minutes, and all groups were further cultured at 37 °C for 24 h. A control group without NIR exposure was included for comparison. After incubation, bacterial suspensions were serially diluted and spread onto agar plates (n=3 per group), which were incubated at 37°C for 24 hours. Images of bacterial colonies were captured. The same procedure was performed with *E. coli* suspension (2.2 × 10⁸ CFU/mL). Bacterial viability was assessed by counting CFUs, and the antibacterial ratio was calculated as: [(Nc − Nt)/Nc] × 100%, where Nc is the control group’s colony count, and Nt is that of the treatment group.

To further clarify the sterilization mechanism of the prepared sponges, SEM was used to observe morphological alterations in bacteria post-treatment. Briefly, treated bacterial cells were harvested by centrifugation, rinsed 4 times with sterile PBS to remove non-adherent substances, and fixed overnight at 4°C in a 2.5% (v/v) glutaraldehyde solution. The fixed bacteria were subsequently subjected to serial dehydration with gradient ethanol solutions (30%, 50%, 70%, 80%, 90%, and 100%, v/v), with each gradient treatment lasting 15 min. After dehydration, the samples were sputter-coated with platinum to enhance electrical conductivity and observed under SEM.

1.5.2 Bacterial membrane integrity assay

The permeability and integrity of the bacterial cell membrane were assessed by monitoring the release of intracellular contents. Following the same treatment protocol as the in vitro antibacterial test (with and without NIR irradiation), the bacterial suspension was centrifuged at 10,000 g for 5 minutes at 4°C. The supernatant containing leaked intracellular substances was carefully collected. The absorbance of the collected supernatant was measured at 405 nm using a UV-Vis spectrophotometer, and the resulting value was used to quantify the released intracellular components, indicating the extent of bacterial membrane damage.

1.5.3 Biofilm eradication assay

The biofilm elimination capability of the sponges was quantitatively assessed using the crystal violet (CV) staining method. *E. coli* and *S. aureus* were, respectively, inoculated into 24-well plates and cultured for 48 h to allow the formation of mature biofilms. Following PBS washing to eliminate planktonic bacteria, the established biofilms were incubated with NS, CuPNS, and TB@CuPNS sponges for 24 h, either with or without 10 min of 808 nm NIR laser irradiation. Subsequently, each well was rinsed with sterile PBS to eliminate unattached cells. The remaining biofilms were fixed in methanol for 15 min and then stained with a 0.1% (w/v) CV solution for 30 min. The remaining biofilms were dissolved in 33% acetic acid, and the absorbance at 590 nm was recorded using a microplate reader.

1.5.4 Quantitative reverse transcription Polymerase Chain reaction (qPCR)

To explore the molecular mechanism underlying bacterial death induced by sponges, the expression levels of cuproptosis-related genes (DLAT, LIAS, GPX4, and FDX1) were analyzed using quantitative reverse transcription-polymerase chain reaction (RT-qPCR). Briefly, *E. coli* and *S. aureus* were treated with NS, CuPNS, and TB@CuPNS (with and without NIR irradiation) as described in the antibacterial assay. After the designated treatments, total bacterial RNA was isolated with a commercial RNA extraction kit, and its concentration and purity were determined according to the manufacturer's protocol. The extracted RNA was then reverse-transcribed into complementary DNA (cDNA) with the PrimeScript™ RT Reagent Kit. The qPCR amplification was conducted on a real-time PCR system using SYBR® Premix Ex Taq™ II. Relative quantification of target gene expression was performed employing the 2−ΔΔCt method, where the 16S rRNA gene was utilized as an endogenous reference. Details of all primer sequences used in this analysis are given in Table S1.

**1.6 Glutathione (GSH) depletion assay**

To confirm the mechanism of copper ion mediated chemodynamic antibacterial activity, glutathione (GSH) consumption was measured. The experiment was conducted under different conditions (pH 7.4 versus pH 5.5, with and without NIR irradiation). Briefly, 10 mM GSH solutions were prepared in PBS buffered to either pH 7.4 or 5.5. CuPNS sponge samples were then added to the respective GSH solutions, and samples in the NIR group were exposed to 808 nm laser irradiation. At designated time intervals, aliquots of the solution were collected, and the concentration of remaining GSH was quantified using a commercial GSH detection kit. The corresponding absorbance was recorded using a microplate reader. A reduction in GSH concentration reflects its consumption by copper ions released from the sponges.

**1.7 Hydroxyl radical generation assay**

Hydroxyl radical (·OH) generation was evaluated using methylene blue (MB) as a chromogenic probe. Briefly, MB solution and H₂O₂ were mixed in PBS at pH 7.4 or 5.5, followed by addition of TB@CuPNS sponges with or without GSH and 808 nm NIR irradiation, according to the experimental grouping. The degradation of MB was monitored by recording the decrease in its characteristic absorbance at 664 nm using a UV-Vis spectrophotometer at predetermined time points. A greater decrease in absorbance at 664 nm indicated stronger MB degradation and higher ·OH-generating catalytic activity.

**1.8 Biocompatibility of the TB@CuPNS in vitro**

1.8.1 In vitro cell proliferation experiment

The viability and proliferation of the L929 cells, HUVECs and HaCaT cells line were evaluated using the live/dead staining method. Cells were incubated with sponge extracts for 24 h, then stained with calcein‑acetoxymethyl ester (calcein‑AM, for live cells) and propidium iodide (PI, for dead cells) in the dark for 30 min. Live and dead cells were visualized by fluorescence microscopy and counted to assess cell viability, with cytotoxicity further evaluated by the CCK-8 assay.

Sponge extracts were prepared according to the following procedure. Composite sponges (NS, CuPNS, TB@CuPNS) were sterilized by immersion in 75% ethanol for 2 h, then rinsed three times with sterile PBS to eliminate residual ethanol. The sterilized sponges were soaked in RPMI‑1640 medium at a concentration of 0.1 g/mL and incubated in a thermostatic shaker (37 °C, 120 rpm) for 24 h. Subsequently, the extracts were filtered through a 0.22 μm sterile filter to ensure sterility and then stored at 4 °C for subsequent experiments. The sponge extracts were incubated for 1, 3, and 5 days, with a blank control group included. After incubation, the culture medium was discarded and substituted with medium supplemented with 10% CCK‑8 reagent. Following a further 30‑min incubation, the absorbance at 450 nm was further measured.

1.8.2 Cell morphology and cytoskeleton staining

Live/dead viability assays of L929 and HUVEC cells cultured in sponge extracts were conducted on days 1, 3, and 5. To visualize cell adhesion and morphology on the sponge surfaces, L929 fibroblasts and HUVECs were stained for the cytoskeleton and cell-specific markers. Sterilized sponge samples were placed in 24-well plates, and cells were seeded directly onto the sponges, then incubated for 48 h. After cultivation, the samples were subjected to immunofluorescence staining, and the corresponding intensities were quantified using ImageJ.

For cytoskeleton staining of L929 cells, sponge constructs seeded with cells were rinsed with sterile PBS, fixed in 4% paraformaldehyde for 15 min, and treated with 0.1% Triton X-100 for 10 min to permeabilize the cell membrane. Following blocking with 5% bovine serum albumin (BSA) for 1 h, fluorescent phalloidin conjugate was applied for 1 h in the dark to label F‑actin filaments. For HUVEC staining, identical fixation, permeabilization, and blocking procedures were adopted. After incubation, the cells were treated overnight at 4 °C with a primary antibody against CD31, an endothelial-specific marker. Following rinsing, fluorescent secondary antibody and phalloidin were applied in the dark for 1 h. All samples were then subjected to nuclear counterstaining with DAPI for 5 min, and the immunofluorescent images were captured.

1.8.3 Scratch Assay

A specific amount of L929 cells was introduced into 6-well plates and maintained in an appropriate culture environment until they covered 90% of the plate surface. Subsequently, a 1 mL pipet tip was carefully taken to generate a scratch on the cell monolayer. After that, cells that had been dislodged were washed away with PBS to remove them. Subsequently, sponge extracts (NS, CuPNS, and TB@CuPNS) were placed into the wells, and cells were cocultured for 24 and 48 h. Cell migration was observed at three time points (0, 24, and 48 h) using fluorescence microscopy. Migration rates were quantified using ImageJ software.

1.8.4 Transwell Assay

The migration capacity of HaCaT cells from different sponge groups was determined by the transwell migration assay. Briefly, a total of 2 × 10^4^ cells were seeded in the upper chamber . The lower chamber was injected with extracts (NS, CuPNS, and TB@CuPNS) separately. After incubating for 24 h, the cells on the upper surface of the membrane were removed by cotton swabs. The cells on the lower surface of the membrane were fixed by 4% PFA and stained with 0.1% crystal violet solution. The stained samples were imaged using a light microscope , and the number of migrated cells was quantified via ImageJ software.

**1.9 In vitro angiogenesis**

The capacity of the sponges to promote angiogenesis was assessed by measuring the formation of tubular structures in HUVECs cultured on Matrigel. Briefly, µ-Slide angiogenesis plates were coated with Matrigel and incubated at 37°C for 30-60 min to allow for polymerization. HUVECs were plated on the pre-polymerized Matrigel at a seeding density of 2 × 10⁴ cells per well, followed by cultivation with medium supplemented with extracts from NS, CuPNS, and TB@CuPNS groups. After incubation for 6-8 h, tubular network formation was monitored and recorded using an inverted optical microscope.

To quantify the extent of angiogenesis, the total branching length and the number of junctions of the capillary-like structures were analyzed from the captured images using the Angiogenesis Analyzer plugin for ImageJ.

**1.10 Antioxidant and anti-inflammatory properties**

1.10.1 Antioxidant property

To assess the sponge's effect on cell viability under oxidative stress conditions, H₂O₂ was used to establish an oxidative stress model. L929 cells, HUVECs, and RAW264.7 macrophages were plated into 96‑well plates at a density of 1 × 10⁴ cells per well and cultured for 24 h to facilitate cell adhesion. The cells were subsequently exposed to 300 µM H₂O₂ prepared in RPMI‑1640 medium for 30 min to establish an oxidative stress model. Then, the cells were co-cultured with the sponge extracts overnight. Cell viability was determined via the CCK‑8 assay and live/dead staining kit (Calcein‑AM and PI), following the protocols.

The antioxidant ability of the TB@CuPNS sponge was further assessed by 2,2-diphenyl-1-picrylhydrazyl (DPPH) radical scavenging assays. NS, CuPNS, and TB@CuPNS samples were introduced into a methanol solution containing 5 μg/mL DPPH (Macklin, Shanghai, China), with the sponge concentrations ranging from 0.01 to 0.1 mg/mL. After incubation at 37 °C in the dark for 30 min, the absorbance at 520 nm was measured by ultraviolet spectrophotometry.

To detect intracellular ROS levels, L929 cells, HUVECs, and RAW264.7 macrophages were seeded at 4 × 10⁴ cells per well into 24‑well plates and incubated for 24 h. After exposure to a 300 μM H₂O₂ solution for 30 min to trigger ROS generation, the cells were washed three times with PBS and further incubated with sponge extracts for 30 min. The cells were then stained with 2',7'-dichlorofluorescin diacetate (DCFH‑DA) in the dark for 30 min, and unbound dye was removed by additional PBS washing. Intracellular ROS levels were visualized using a fluorescence microscope (Olympus IX71, Japan).

1.10.2 Anti-inflammatory property

Raw264.7 macrophages were plated into 96-well plates and cultured for 24 h to allow cell adhesion. The cells were then co-cultured with NS, CuPNS, and TB@CuPNS sponge extracts at concentrations of 1 and 5 μg/mL for 24 h, followed by stimulation with 0.5 μg/mL lipopolysaccharide (LPS) to induce an inflammatory response. After receiving LPS stimulation for 24 h, the cells were immersed in 4% (w/v) paraformaldehyde at ambient temperature for 10 min, then incubated with 10% (v/v) goat serum for 1 h to reduce non‑specific immunoreactions.

Subsequently, the cells were separately incubated overnight with an iNOS antibody (1:300, ab49999, Abcam, Cambridge, UK) and an Arg1 antibody (1:300, ab272660, Abcam, Cambridge, UK). After being washed three times with PBS, the cells were treated with Cora Lite 488‑labeled goat anti-rabbit secondary antibody (1:800, SA00013‑2, Proteintech, Wuhan, China). After counterstaining the nuclei with DAPI (ab104139, Abcam, Cambridge, UK) for 5 min, immunofluorescent images were captured using a fluorescence microscope.

**1.11 PCR real-time quantitative polymerase chain reaction**

L929 cells, HUVECs, and RAW264.7 macrophages cultured on the sponges were collected for total RNA extraction using an RNA purification kit (ES Science, Shanghai, China). RNA concentration and purity were determined using a NanoDrop spectrophotometer. Reverse transcription of the extracted RNA into cDNA was carried out using a commercial kit (ABM, Vancouver, Canada). qRT‑PCR was subsequently conducted on a CFX96™ Real‑Time PCR System (Bio‑Rad, Hercules, CA, USA) with SYBR Green qRT‑PCR Master Mix (TOYOBO, Osaka, Japan).

Relative gene expression levels were quantified using the 2−ΔΔCt approach, using GAPDH for normalization. The transcript levels of key genes associated with tissue regeneration and inflammatory modulation were determined. These analyses were performed to evaluate the immunomodulatory activity and pro‑regenerative capacity of the prepared sponges. The detailed primer sequences utilized are listed in Table S2.

**1.12 In vitro hemostasis experiments**

1.12.1 Blood compatibility

Fresh anticoagulated blood (1 mL) was combined with 4 mL of sterile PBS and centrifuged at 250 g for 15 min to collect erythrocytes. The resulting erythrocyte precipitate was resuspended in 9 mL of sterile PBS. Sponge specimens (2 mm height, 3 mm diameter) were placed in 24-well plates, each well receiving 1 mL of the erythrocyte suspension. Following incubation at 37 °C for 1 h, the samples were centrifuged at 2000 g for 15 min. A microplate reader was employed to determine the absorbance of the supernatant at 540 nm. Sponge specimens (2 mm height, 3 mm diameter) were placed in 24-well plates, each well receiving 1 mL of the erythrocyte suspension. The hemolysis ratio was calculated according to the following equation: Hemolysis ratio (%) = (A_Experiment_ − A_Negative_) / (A_Positive_ − A_Negative_) × 100 %, where A_Experiment_, A_Negative_, and A_Positive_ represent the absorbance values of the experimental, negative control, and positive control groups, respectively.

1.12.2 Red blood cells and platelet adhesion assays

In the red blood cell (RBC) adhesion test, 200 µL of anticoagulated fresh whole blood was added to 24-well plates containing 10 mg of each specimen, and the plates were incubated at 37 °C for 30 min. After incubation, non-specifically bound RBCs were removed by rinsing the sample surface three times with PBS. The specimens were subsequently fixed with 2.5% paraformaldehyde for 3 h, then washed with PBS to eliminate residual fixative, and dehydrated sequentially in ethanol solutions with gradient concentrations (20%, 40%, 60%, 80%, and 100%). After air-drying at room temperature, the adhesion of RBCs to the sponge surface was observed using SEM.

For the platelet adhesion assay, fresh venous blood obtained from rabbits was centrifuged at 3000 rpm for 15 min to prepare platelet-rich plasma (PRP). PRP droplets were added to different sponge samples placed in 24-well plates and incubated at 37 °C for 30 min. After incubation, non-adherent platelets were eliminated by rinsing the samples three times with PBS. The specimens were fixed overnight at 4 °C in a 2.5% (v/v) glutaraldehyde solution, then serially dehydrated in 20%, 40%, 60%, 80%, and 100% (v/v) ethanol, and dried at 37 °C. The adhesion and morphology of platelets on the sponge surface were observed using SEM.

**1.13 In vivo hemostatic experiments**

A rat liver bleeding model was performed to evaluate the hemostatic abilities of NS, CuPNS, and TB@CuPNS sponges. Sprague-Dawley (SD) rats were anesthetized with isoflurane, and a midline abdominal incision was made to expose the liver. A 1 cm length and 2 mm in depth wound was made on the liver surface using a scalpel to induce bleeding. Subsequently, NS, CuPNS, and TB@CuPNS sponge samples (2 mm height, 15 mm diameter) were compressed and promptly applied to the bleeding region. A blank control group was included for comparative analysis, and both bleeding time and blood loss volume were quantitatively recorded.

A rat femoral artery puncture bleeding model was also used to further evaluate hemostatic efficacy. At the location close to the femoral artery, a midline skin incision about 1 centimeter long was made, and then the femoral artery was exposed by separating the surrounding connective tissue. Puncture of the femoral artery was performed with a 10 mL syringe needle to induce bleeding, and the sponge samples (NS, CuPNS, TB@CuPNS) or blank control were immediately applied to the bleeding area, gently pressed to ensure full contact with the wound under suitable pressure. The blood loss and hemostatic time were recorded. No treatment after bleeding served as the blank control group.

**1.14 In vivo wound healing experiment**

All in vivo experimental procedures implemented in this research were conducted in compliance with the relevant ethical standards and have been authorized by the Animal Ethics Committee of Peking University People’s Hospital. The diabetes model was established via intraperitoneal injection of streptozotocin (STZ) , and only those mice with persistently elevated blood glucose levels exceeding 16.7 mmol/L were selected for subsequent experiments. These diabetic mice were randomly assigned into five experimental groups with 15 mice per group (n=15): Control (PBS) group, NS group, CuPNS group, TB@CuPNS group, and TB@CuPNS + NIR group. For each group, 15 mice were allocated across the experimental time points, including 3 mice for day 3 analysis, 6 mice for day 7 analysis, and 6 mice for day 14 analysis. Wound-closure analysis, bacterial burden assessment, histological/IHC staining, RNA sequencing, and qRT-PCR validation were performed using samples collected at the corresponding time points, with the exact sample size for each assay indicated in the figure legends. After the mice were anesthetized and shaved, a 10-millimeter full-thickness skin wound was created, and 3.5 × 10⁶ CFU of *S. aureus* was inoculated. The infected wounds were treated with 10 µL of PBS or the respective sponge sample. For the TB@CuPNS + NIR group, the wound was exposed to an 808 nm NIR laser (0.7 W·cm⁻²) for 10 min immediately after sample application, and infrared thermographic images were recorded during irradiation.

For histological examination, the excised wound tissue was fixed in 4% paraformaldehyde, embedded in paraffin wax, and sectioned. Hematoxylin and Eosin (H&E) staining was performed on the sections to assess the overall tissue morphology, while Masson's trichrome staining was employed to detect collagen fiber deposition. To more clearly visualize marker localization together with tissue architecture, immunohistochemistry (IHC) staining was performed on paraffin-embedded wound sections. Specific markers, including TNF-α, IL-6, CD86, CD206, CD31, α-SMA, Collagen I, and Collagen III, were employed for this purpose. IHC staining was quantified using ImageJ, and the positive staining area or mean density was calculated as indicated in the corresponding figure legends. For RNA-sequencing assays, total RNA was isolated from wound tissue, after which libraries were then prepared and sequenced using the Illumina platform to identify differentially expressed genes (DEGs) and critical biological pathways.

For qRT-PCR validation in animal wound tissues, epithelial repair-related genes (Krt14, Krt16, Grhl3, and Lamb3) and inflammation/chemokine-related genes (Mmp12, Marco, Ccl5, and Cxcl10) were analyzed to validate the RNA-seq-derived wound-repair and inflammatory-regulation signatures. Relative expression was calculated using the 2−ΔΔCt method. Primer sequences are listed in Table S3.

**2. Results**

**Figure S1.** Nanofiber diameter of different sponges.

**Figure S2.** Compression cyclic Young’s modulus of the TB@CuPNS sponge with different cycles.

**Figure S3.** Quantification of sponge porosity in different groups.

**Figure S4.** Photothermal temperature change curves of the NS and CuPNS sponges under different laser power densities. (A) Temperature profiles of NS and CuPNS sponges in the dry state under 808 nm NIR irradiation at 0.05, 0.3, and 0.7 W·cm⁻². (B) Temperature profiles of NS and CuPNS sponges in moist conditions under 808 nm NIR irradiation at 0.05, 0.3, and 0.7 W·cm⁻² for 10 min.

**Figure S5.** CCK-8 assay evaluating the biocompatibility of different copper ion concentrations. Statistical analysis was performed using one-way ANOVA with Tukey’s post hoc test. n = 3 independent experiments per group. All data in the graphs are presented as mean ± SD; *P < 0.05, **P < 0.01.

**Figure S6.** CCK-8 assay of HaCaT keratinocytes treated with extracts from different sponge groups. Statistical analysis was performed using one-way ANOVA with Tukey’s post hoc test. n = 3 independent experiments per group. All data in the graphs are presented as mean ± SD; *P < 0.05 and **P < 0.01 versus the NS group; #P < 0.05 and ##P < 0.01 versus the CuPNS group.

Figure S7. Scratch wound healing assay evaluating L929 fibroblast migration after treatment with different sponge extracts. (A) Scratch wound healing assay with different treatments on L929 cells at 0, 24, and 48 h. Scale bar = 200 μm. (B) Quantitative analysis of L929 cell migration rate in different groups. Statistical analysis was performed using one-way ANOVA with Tukey’s post hoc test. n = 3 independent experiments per group. All data in the graphs are presented as mean ± SD; *P < 0.05 and **P < 0.01 versus the NS group; #P < 0.05 and ##P < 0.01 versus the CuPNS group.

Figure S8. Transwell migration assay of HaCaT keratinocytes treated with different sponge extracts. (A) Transwell assay showing the migration ability of HaCaT cells cultured in different groups. Scale bar = 100 μm. (B) Quantitative analysis of migrated HaCaT cells in different groups. Statistical analysis was performed using one-way ANOVA with Tukey’s post hoc test. n = 3 independent experiments per group. All data in the graphs are presented as mean ± SD; *P < 0.05, **P < 0.01.

**Figure S9.** Representative photographs showing the wound-size measurement markers used to quantify wound closure during the in vivo diabetic infected wound healing experiment.

**Figure S10.** Quantification of wound closure rates from different groups on days 3, 7, and 14. Statistical analysis was performed using one-way ANOVA with Tukey’s post hoc test. n = 6 animals per group. All data in the graphs are presented as mean ± SD; *P < 0.05, **P < 0.01.

**Figure S11.** In vivo antibacterial rate against *S. aureus* in wound tissues from different treatment groups. Statistical analysis was performed using one-way ANOVA with Tukey’s post hoc test. n = 6 animals per group. All data in the graphs are presented as mean ± SD; **P < 0.01.

**Figure S12.** Quantitative analysis of inflammatory and macrophage-polarization markers in wound tissues**.** Quantification of relative positive area of (A) CD86，(B) CD206，(C) TNF-α，and (D) IL-6 in different treatment groups. Statistical analysis was performed using one-way ANOVA with Tukey’s post hoc test. n = 3 animals per group. All data in the graphs are presented as mean ± SD; **P < 0.01.

**Figure S13.** H&E staining images of wound tissues from different groups on day 7 (red arrows: granulation tissue gap; red stars: regenerated hair follicles).

**Figure S14.** Representative Masson’s trichrome staining images of wound tissues from different groups on day 7 (blue arrows: wound center).

**Figure S15.** Quantification of wound healing length from different groups on days 7 and 14. Statistical analysis was performed using one-way ANOVA with Tukey’s post hoc test. n = 6 animals per group. All data in the graphs are presented as mean ± SD; *P < 0.05, **P < 0.01.

**Figure S16.** Quantification of newly formed granulation tissue thickness from different groups on days 7 and 14. Statistical analysis was performed using one-way ANOVA with Tukey’s post hoc test. n = 6 animals per group. All data in the graphs are presented as mean ± SD; *P < 0.05, **P < 0.01.

**Figure S17.** Representative IHC staining images of CD31 in wound tissues from different treatment groups on days 7 and 14.

**Figure S18.** Quantification of CD31-positive staining area in wound tissues from different treatment groups on days 7 and 14. Statistical analysis was performed using one-way ANOVA with Tukey’s post hoc test. n = 6 animals per group. All data in the graphs are presented as mean ± SD; **P < 0.01.

**Figure S19.** Representative H&E-stained images of major organs (heart, liver, spleen, lungs, and kidneys) from different treatment groups.

**Figure S20.** qRT-PCR validation of RNA-seq-derived epithelial repair and re-epithelialization genes in wound tissues. Krt14, Krt16, Grhl3, and Lamb3 were selected to represent keratinocyte activation, epithelial differentiation, barrier repair, and basement-membrane reconstruction. Statistical analysis was performed using one-way ANOVA with Tukey’s post hoc test. n = 3 independent experiments per group.

All data in the graphs are presented as mean ± SD; **P < 0.01, ***P < 0.001, ****P < 0.0001.

**Figure S21.** qRT-PCR validation of RNA-seq-derived inflammation- and chemokine-related genes in wound tissues. Mmp12, Marco, Ccl5, and Cxcl10 were selected to represent macrophage-associated remodeling and inflammatory chemokine signaling. Statistical analysis was performed using one-way ANOVA with Tukey’s post hoc test. n = 3 independent experiments per group. All data in the graphs are presented as mean ± SD; **P < 0.01, ***P < 0.001.

**Table S1** The primers for the target genes

| Species | Gene | Primer sequences (5’-3’) | |
| --- | --- | --- | --- |
| *S.*  *aureus* | DLAT | Forward | GCAGCAGTATCATTAGAAGGCGA |
|  |  | Reverse | TTCTTACGGTGATCCCATAATGC |
|  | GPX4 | Forward | TGCGTAAATAACGGATGTTCATC |
|  |  | Reverse | ATCGTGGGTTTGTAGTGTTGAGT |
|  | FDX1 | Forward | ATGGATACTTGTATTGCATGTGGTG |
|  |  | Reverse | TAAAGCGTCCCCATCAAATGAT |
|  | LIAS | Forward | ACCACTTAAATCGGGTCAAGAAC |
|  |  | Reverse | GAATGCTTGTTTAGCGATGGA |
|  | 16s rRNA | Forward | ACAATACAAAGGGCAGCGAAAC |
|  |  | Reverse | CGATACGGCTACCTTGTTACGAC |
| *E. coli* | DLAT | Forward | ATGATCCCGCATGTTACCCA |
|  |  | Reverse | TCATGATGAAGACAACTGGGGTT |
|  | GPX4 | Forward | TTAGGATCTGTCTTTGAGGAATAGC |
|  |  | Reverse | ACACTACTTTCCCTCGCTTTGCT |
|  | FDX1 | Forward | CGATTTCGATGGGTGAACATA |
|  |  | Reverse | TTATCCCACAACTGTTCTTCCGT |
|  | LIAS | Forward | CTGAGTACGAATAGCAGTGATGGTC |
|  |  | Reverse | GCTGAACGGTTGCTGGAAA |
|  | 16s rRNA | Forward | AGGTGTAGCGGTGAAATGCG |
|  |  | Reverse | CATCGTTTACGGCGTGGACT |

**Table S2** Primer sequences of the genes for qRT-PCR analysis

| Gene | Primer sequences (5’-3’) | |
| --- | --- | --- |
| GAPDH | Forward | GAAGGGCATCTTGGGCTACAC |
|  | Reverse | GTTGTCATTGAGAGCAATGCCA |
| Col I | Forward | TAGAGGCTCTGAAGGTCCCC |
|  | Reverse | CACCAGCAATACCAGGAGCA |
| Col III | Forward | CAGCCAGGTCGAGATGGATC |
|  | Reverse | CAGGGCCAGTTTCTCCTCTG |
| VEGF | Forward | TCTCTACCCCAGGTCAGACG |
|  | Reverse | CCCAAAGCACAGCAATGTCC |
| eNOS | Forward | TGTCCAACATGCTGCTGGAAATTG |
|  | Reverse | AGGAGGTCTTCTTCCTGGTGATGCC |
| Akt | Forward | CGAGCTGTTCTTCCACCTGT |
|  | Reverse | GTTCTCCAGCTTGAGGTCCC |
| TNF-α | Forward | GGCAGGTTCTGTCCCTTTCA |
|  | Reverse | TCTTCTGCCAGTTCCACGTC |
| iNOS | Forward | GCCAACATGCTACTGGAGGT |
|  | Reverse | TCCAGGATGTTGTAGCGCTG |
| IL-10 | Forward | CCAAGCCTTATCGGAAATGA |
|  | Reverse | TCCTGAGGGTCTTCAGCTTC |
| Arg1 | Forward | TTTCTCAAAAGGACAGCCTCG |
|  | Reverse | ACAGACCGTGGGTTCTTCAC |

Table S3 Primer sequences used for qRT-PCR validation of RNA-seq-derived wound-repair and inflammatory genes in animal wound tissues.

| Gene | Primer sequences (5’-3’) | |
| --- | --- | --- |
| GAPDH | Forward | GAAGGGCATCTTGGGCTACAC |
|  | Reverse | GTTGTCATTGAGAGCAATGCCA |
| Krt14 | Forward | CCCCTACTTCAAGACCATCGAG |
|  | Reverse | CTGCTCCGTCTCAAACTTGGTC |
| Krt16 | Forward | GGTGGCCTCTAACAGTGATCT |
|  | Reverse | TGCATACAGTATCTGCCTTTGG |
| Grhl3 | Forward | ACAGAGGAGGTGTTTGACGC |
|  | Reverse | CATGTTAACCAGGATGCCTCGC |
| Lamb3 | Forward | GGGATCTGGAGAAAGGCAGG |
|  | Reverse | GAAGCCAGGCAAGGCAAG |
| Mmp12 | Forward | TTGCATTTGGAGCTCACGGA |
|  | Reverse | CCCAAGGAATGGCCAAGTTCA |
| Marco | Forward | TGCAGGTGTGAAAGGAAGCA |
|  | Reverse | TCTGCCTACAAGACCTGGGA |
| Ccl5 | Forward | GCCCTCACCATCATCCTCAC |
|  | Reverse | CGAGTGACAAACACGACTGC |
| Cxcl10 | Forward | GATGACGGGCCAGTGAGAAT |
|  | Reverse | CATCGTGGCAATGATCTCAACA |

**Supplemental Movies**

Video S1. Shape recovery and water absorption behavior of the TB@CuPNS sponge after contact with aqueous solution.

Video S2. Structural stability of the TB@CuPNS sponge under vigorous mechanical stirring in aqueous solution.

Video S3. Reversible compression and recovery behavior of the TB@CuPNS sponge under repeated external deformation.
